# Low-Abundance and Fragmentary *Helicobacter pylori* DNA Detected in Phenotypically Negative Gastric Biopsies Using Targeted Sequencing

**DOI:** 10.3390/biom16060765

**Published:** 2026-05-22

**Authors:** Fabien Mbaya-Tshibangu, Alain Cimuanga-Mukanya, Evariste Tshibangu-Kabamba, Nadine Kayiba-Kalenda, Tressy Kalenga-Ngomba, Patrick de Jesus Ngoma-Kisoko, Gunturu Revathi, Junko Akada, Benoît Mbiya-Mukinayi, Augustin Tshibaka Kabongo, Ghislain Disashi-Tumba, Takashi Matsumoto, Yoshio Yamaoka

**Affiliations:** 1 Department of Environmental and Preventive Medicine, Faculty of Medicine, Oita University, Yufu 879-5593, Japan; m22d9106@oita-u.ac.jp (F.M.-T.);; 2 Department of Internal Medicine, Faculty of Medicine, Pharmacy and Public Health, University of Mbujimayi, Mbujimayi P.O. Box 225, Congo; vdefacmed@um.ac.cd (A.C.-M.); sec_gen_recherche@um.ac.cd (E.T.-K.);; 3 Department of Virology and Parasitology, Research Center for Infectious Disease Sciences, Graduate School of Medicine, Osaka Metropolitan University, Osaka 545-8585, Japan; 4 Department of Public Health, Faculty of Medicine, Pharmacy and Public Health, University of Mbujimayi, Mbujimayi P.O. Box 225, Congo; 5 Research Institute of Health and Society, Catholic University of Louvain, 1200 Brussels, Belgium; 6 Department of Internal Medicine, Faculty of Medicine, University of Kinshasa, Kinshasa P.O. Box 834, Congo; 7 Department of Clinical Microbiology, Aga Khan University Hospital, Nairobi 00100, Kenya; 8 Department of Paediatrics, Faculty of Medicine, Pharmacy and Public Health, University of Mbujimayi, Mbujimayi P.O. Box 225, Congo; 9 Department of Global Health, School of Tropical Medicine and Global Health, Nagasaki University, Nagasaki 852-8523, Japan; 10 Research Center for Global and Local Infectious Diseases, Oita University, Yufu 879-5593, Japan; 11 Department of Medicine, Gastroenterology and Hepatology Section, Baylor College of Medicine, Houston, TX 77030, USA; 12 Division of Gastroentero-Hepatology, Department of Internal Medicine, Faculty of Medicine-Dr. Soetomo Teaching Hospital, Universitas Airlangga, Surabaya 60286, Indonesia

**Keywords:** *H. pylori*, antimicrobial resistance, long-read sequencing, selective whole-genome amplification, phenotypically negative biopsies, low-biomass samples, natural transformation

## Abstract

Accurate detection and monitoring of antimicrobial resistance (AMR) in *Helicobacter pylori* mainly rely on phenotypic methods and culture, which can sometimes fail when bacterial load is low or after recent treatment. We investigated whether gastric biopsies classified as *H. pylori*-negative by standard diagnostic techniques still contain detectable bacterial DNA, including regions linked to AMR, and assessed whether selected DNA fragments can mediate allelic exchange in vitro. Gastric biopsies from 46 dyspeptic patients in the Democratic Republic of the Congo (including 23 phenotypically positive and 23 phenotypically negative individuals) were analyzed using long-read amplicon sequencing of seven resistance-associated loci, selective whole-genome amplification (sWGA) followed by long-read sequencing of *H. pylori*-enriched reads, and a proof-of-concept natural transformation assay. Phenotypically negative biopsies exhibited significantly lower sequencing depth across multiple loci (including *23S rRNA*, *gyrA*, *gyrB*, and *pbp1A*; *p* = 0.003–0.014), indicating a reduced *H. pylori* DNA burden. However, AMR-associated mutations linked to various antibiotic classes were found in both groups. sWGA enabled recovery of fragmentary *H. pylori* sequence data from phenotypically negative samples, including reads that map to resistance- and virulence-associated genes. In vitro, *23S rRNA* A2143G amplicons from both phenotypically positive and negative biopsies produced clarithromycin-resistant transformants in strain 26695. These findings indicate that phenotypically negative gastric biopsies might contain low-abundance and fragmentary *H. pylori* DNA. Although certain DNA fragments can mediate allelic exchange under controlled in vitro conditions, these results do not confirm bacterial viability, active infection, or clinically relevant in vivo resistance transfer. Therefore, they should be interpreted with caution in molecular AMR surveillance and detection contexts.

## 1. Importance

Routine diagnostics and antimicrobial resistance (AMR) surveillance for *H. pylori* mainly depend on phenotypically positive patients. Our findings indicate that gastric biopsies from phenotypically negative patients may still contain low levels or fragmented *H. pylori* DNA, including AMR-related genes. Therefore, restricting analysis to positive samples could result in incomplete detection and biased interpretation of AMR patterns. However, the presence of AMR-associated molecular signals in these samples should not be interpreted as evidence of active infection or clinically significant AMR, as they likely represent residual or low-abundance bacterial material that falls below the detection limits of standard methods. Methodologically, these findings emphasize the importance of cautious interpretation of molecular data in low-biomass samples, taking into account sequencing depth, gene recovery, and amplification biases. Overall, combining sensitive molecular techniques with rigorous controls and quantitative frameworks may enhance the accuracy and completeness of AMR surveillance, even in specimens that are classified as negative by traditional diagnostic methods.

## 2. Introduction

*Helicobacter pylori* is one of the most common bacterial infections in humans, colonizing the stomach lining. It causes chronic gastritis, peptic ulcers, gastric MALT lymphoma, and gastric adenocarcinoma [1]. Due to its proven carcinogenic role, it has been classified as a Group 1 carcinogen by the International Agency for Research on Cancer (IARC) since 1994 [2]. Without an effective vaccine, eradication therapy remains the primary treatment, helping to resolve symptoms and prevent gastric cancer. However, rising antimicrobial resistance (AMR) has become a significant obstacle to successful treatment, underscoring the need to better understand *H. pylori* resistance mechanisms [3].

In *H. pylori*, antimicrobial resistance has traditionally been linked to chromosomal mutations that arise through vertical gene transfer [4,5]. The gastrointestinal tract acts as a major reservoir of antimicrobial resistance genes (ARGs), promoting their exchange among bacterial populations [6]. However, the role of HGT remains poorly understood, despite the organism’s natural ability to absorb external DNA via natural transformation [7,8,9], suggesting that exogenous or residual DNA fragments in the gastric environment may facilitate genetic exchange.

Several pathways can introduce *H. pylori*-related ARGs into the gastric environment, including contaminated food and water, transient exposure to resistant bacteria [10], and prior or partially treated infections [11]. These pathways may be more common in high-prevalence regions like sub-Saharan Africa, where antibiotic use is also widespread [12]. At the same time, traditional diagnostic methods for *H. pylori* are prone to false-negative results, especially in cases of low bacterial load, patchy gastric colonization, or recent exposure to antibiotics, bismuth compounds, or proton pump inhibitors [13,14,15]. As a result, dyspeptic patients classified as *H. pylori*-negative may still harbor low levels of bacterial DNA or potentially viable but metabolically inactive, non-culturable organisms within the gastric mucosa [16]. Additionally, antimicrobial susceptibility testing mainly depends on culture-positive isolates, which systematically excludes phenotypically negative patients from routine resistance monitoring [17]. These factors create a diagnostic blind spot that may limit the completeness of antimicrobial resistance (AMR) surveillance and complicate the interpretation of resistance patterns based only on diagnostic-positive samples, especially in high-burden settings such as the Democratic Republic of the Congo (DRC). However, it remains uncertain how much such undetected *H. pylori* genetic material contributes to clinically or epidemiologically significant AMR.

Interpreting molecular signals in low-biomass environments remains difficult. Studies in low-biomass microbiology indicate that sequencing-based detection can be affected by background contamination introduced during DNA extraction and amplification, especially when target DNA is at very low levels. Additionally, the detected DNA might come from non-viable bacteria, extracellular DNA, or transient exposure rather than active colonization. As a result, distinguishing true biological signals from artifacts requires careful experimental design, proper controls, and cautious interpretation of results [18,19]. These issues are particularly challenging when evaluating antimicrobial resistance-related loci, where detection does not always mean clinical relevance.

Molecular diagnostic approaches, including conventional PCR, real-time PCR, and digital droplet PCR, provide enhanced sensitivity for detecting *H. pylori* and known resistance determinants [17]. However, these methods are limited to predefined targets, offer minimal genomic context, and do not capture the full diversity of ARGs [20]. Targeted PCR amplicon sequencing offers an intermediate approach, allowing high-resolution characterization of resistance loci and linking mutations within genetic backgrounds, but remains confined to specific genomic regions [21]. Selective whole-genome amplification (sWGA) is a complementary, culture-independent method that enriches pathogen DNA from host-dominated clinical samples, including low-abundance or fragmented templates. This technique has facilitated genome recovery for pathogens such as *Mycobacterium*, *Leishmania*, and *Plasmodium* without the need for culture [22,23,24]. However, sWGA is also prone to amplification bias and uneven genome recovery, especially in low-biomass samples, and has not yet been applied to *H. pylori*.

While previous studies have reported the detection of *H. pylori* DNA in samples classified as negative by conventional diagnostic methods, most have depended on a single molecular technique and offer limited characterization of the recovered genetic material [17,21,25]. This study builds on that work by combining targeted long-read amplicon sequencing, selective whole-genome amplification, and proof-of-concept in vitro transformation assays within a single experimental setup. This integrated approach not only detects low-abundance molecular signals but also characterizes resistance-related loci and evaluates whether the selected DNA fragments retain enough sequence integrity for experimental transformation in a controlled laboratory setting. Collectively, these methods represent a significant advancement for studying *H. pylori*-associated molecular signals in clinical samples with relatively low biomass.

In this study, we developed targeted long-read amplicon sequencing and sWGA-based long-read sequencing to enable culture-independent recovery of low-abundance or fragmented *H. pylori* DNA from gastric biopsy specimens collected from individuals who were phenotypically positive and negative in the DRC. We also conducted in vitro natural transformation assays as a proof-of-concept to assess whether selected resistance-associated *23S rRNA* DNA fragments from phenotypically negative biopsies maintained enough sequence integrity to facilitate homologous recombination under laboratory conditions. Our goal was not only to detect *H. pylori* DNA in phenotypically negative biopsies but also to interpret such low-abundance molecular signals within antimicrobial resistance surveillance. Overall, this work provides a cautious framework for understanding molecular AMR signals in low-biomass samples and for designing more comprehensive AMR surveillance strategies.

## 3. Methods

### 3.1. Study Design and Sample Selection

**Figure 1:**
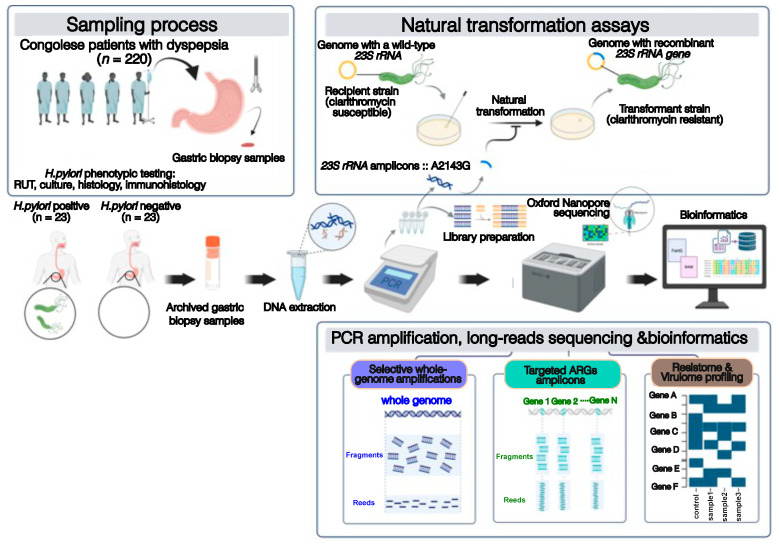
Overall study framework. Schematic overview of the study design, including patient recruitment, gastric biopsy collection, targeted amplification of AMR-associated loci, and long-read sequencing. Downstream analyses included determining the phenotypic status of *Helicobacter pylori*, assessing sequencing signals (read depth), and profiling gene recovery. The workflow also shows the in vitro natural transformation experiments conducted to evaluate whether AMR-associated DNA fragments recovered from gastric biopsies of both phenotypically *H. pylori*-positive and -negative patients maintained enough integrity to mediate allelic exchange under in vitro conditions.

We examined gastric biopsy specimens collected from patients with dyspepsia in the Democratic Republic of the Congo (DRC). These samples came from archived specimens obtained during a multicenter cross-sectional survey conducted between 2017 and 2019 at four healthcare facilities in Kinshasa, DRC (see Figure 1). Patients who had recently used antibiotics, bismuth compounds within the past 4 weeks, or proton pump inhibitors (PPIs) within the past 2 weeks were excluded from participation. The clinical and demographic details of participants were recorded as part of the main cohort study; however, detailed stratified analyses based on these variables were not performed in this study [26]. In the main cohort of 220 patients who underwent upper gastrointestinal endoscopy, *H. pylori* status was determined using phenotypic diagnostic methods, including on-site rapid urease testing as well as laboratory testing such as culture, histology, and immunohistochemistry, as previously described [26]. Remaining antral biopsy tissue homogenates (prepared in 500 µL sterile phosphate-buffered saline [PBS]) were stored at −80 °C until further testing. Based on the phenotypic results available (culture, rapid urease test, histology, immunohistochemistry) in the main cohort, 109 patients were classified as *H. pylori*-negative.

For the molecular analyses presented here, we chose a balanced subset of 46 archived gastric biopsy specimens, including 23 phenotypically positive and 23 phenotypically negative samples. Phenotypically negative samples were defined as biopsies that had undergone at least three of the four diagnostic tests (rapid urease test, culture, histology, and immunohistochemistry) and tested negative in all assays performed. In contrast, phenotypically positive samples were identified by positivity in at least one conventional diagnostic test. Since phenotypic diagnostic methods can have reduced sensitivity in low-burden infections, especially in samples with low levels of *H. pylori*, classifying samples as phenotypically negative should be considered as an operational definition rather than conclusive evidence that *H. pylori*-related material is completely absent. This balanced approach was designed to compare the detectability and sequencing signal of *H. pylori* resistance-related loci between groups with different phenotypic statuses. The cultured *H. pylori* strain 26695 was used as a positive control to validate the assay.

### 3.2. Biopsy DNA Extraction

Frozen antral biopsy homogenates were thawed on ice, and total genomic DNA was extracted using the DNeasy Blood & Tissue Kit (QIAGEN, Hilden, Germany) following the manufacturer’s instructions. DNA concentrations were adjusted to approximately 2 ng/µL, then samples were further purified with the DNA Clean & Concentrator Kit (Zymo Research, Irvine, CA, USA) to remove residual PCR inhibitors. Extraction blanks, purification blanks, no-template PCR controls, and water/matrix-free controls were included to monitor potential contamination during DNA extraction, purification, and amplification. Samples were considered free of detectable upstream contamination when negative controls showed no measurable DNA signal via Qubit 1X dsDNA HS Assay Kit quantification (Thermo Fisher Scientific, Waltham, MA, USA) and no visible amplification products under the same experimental conditions as test samples. These criteria were applied to extraction blanks, purification blanks, and no-template PCR controls to evaluate potential contamination during upstream processing steps.

### 3.3. PCR Amplification, Sequencing, and Bioinformatics of ARGs

Seven *H. pylori* ARGs were targeted for PCR amplification: *gyrA* and *gyrB* (quinolone resistance) [27,28], *16S rRNA* (tetracycline resistance) [29], *23S rRNA* (macrolide resistance) [30], *frxA* and *rdxA* (metronidazole resistance) [31], as well as *pbp1A* (amoxicillin resistance) [32]. Primers were designed from full-length consensus sequences generated by aligning ARGs from African *H. pylori* strains (multi-sheet Supplementary Data File S1: Afro_Hp_ARGs_sequences) using SnapGene v7.2.1 (Dotmatics, Boston, MA, USA). Primer binding sites were selected from less polymorphic regions near the 5′ and 3′ ends of each gene to maximize coverage across diverse strains. Primer-target alignment characteristics, including BLASTn v2.16.0 match scores, are provided in the multi-sheet Supplementary Data File S2: Afro_ARGs_Primers_Blast_Matches. An in silico evaluation of primer coverage and predicted amplification efficiency across *H. pylori* genomes is presented in Supplementary Table S7. Primer sequences are listed in Supplementary Table S6.

PCR amplification was carried out using PrimeSTAR GXL Premix (Takara Bio Inc., Otsu, Japan) in 50 µL reaction volumes containing 25 µL of 2× premix, approximately 0.4 µM of each primer, and about 5 ng of template DNA. No-template controls were included in each run. The cycling conditions included an initial denaturation at 98 °C for 2 min, followed by 30 cycles of 98 °C for 10 s, 55 °C for 15 s, and 68 °C for 45 to 160 s (depending on amplicon length), with a final extension at 68 °C for 5 min. PCR products were evaluated using the Agilent TapeStation system and purified prior to library preparation.

Amplicon libraries were prepared using the Native Barcoding Kit 24 V14 (SQK-NBD114.24; Oxford Nanopore Technologies, Oxford, UK). Barcoded amplicons were pooled and sequenced on a FLO-MIN114 (R10.4.1) flow cell with a GridION X5 Mk1 platform for a 6 h run.

### 3.4. Selective Whole-Genome Amplification (sWGA) Sequencing and Bioinformatics

To complement targeted-locus sequencing in phenotypically negative biopsies, we selected 11 samples for selective whole-genome amplification (sWGA) to enrich for *H. pylori* DNA. Samples included in the sWGA analysis were selected sequentially after targeted sequencing, without prior knowledge of sequencing results or mutation profiles, to minimize selection bias. The number of samples analyzed by sWGA was limited due to resource constraints; thus, this subset primarily served as a proof-of-concept for enriching and recovering *H. pylori*-derived sequence fragments, rather than for estimating prevalence or conducting comparative statistical analysis. sWGA primer sets were designed using the SOAPswga pipeline [22]. The complete genome sequence of *H. pylori* strain CKIN1, cultured from a Congolese patient, was used as the on-target reference, while the human reference genome assembly GRCh38.p14 (NCBI RefSeq accession: GCF_000001405.40) served as the off-target background. SOAPswga identifies short oligonucleotide motifs (k-mers) that are enriched in the target genome but rare in the background, thereby improving the selective amplification of pathogen DNA from host-dominated samples [22].

SOAPswga generated ten candidate primer sets. We selected one set consisting of five 8-mer primers with the highest predicted specificity and amplification efficiency for experimental validation (AGCGTA*T*T, GCGATA*T*T, TAGCGA*T*C, TAGCGA*T*T, and TTTAGC*G*A; asterisks indicate phosphorothioate linkages between the terminal 3′ nucleotides). Phosphorothioate modifications were added at the last three positions at the 3′ end of each primer to improve resistance to exonuclease activity. Primers were synthesized commercially and used for downstream sWGA reactions [33].

Multiple displacement amplification (MDA) was carried out using phi29 DNA polymerase. Each 50 µL reaction included 10 ng of template DNA, 20 U of phi29 DNA polymerase (New England Biolabs, Ipswich, MA, USA, M0269L), 1× phi29 reaction buffer, 1 mM each dNTP (NEB, Ipswich, MA, USA, N0447S), 0.1 mg/mL bovine serum albumin (NEB, Ipswich, MA, USA, B9200S), and 10 µM of each sWGA primer. Reactions were prepared on ice and underwent a hybridization step with decreasing temperature from 35 °C to 30 °C (1 °C decrements every 10 min), then performed at 30 °C for 16 h for isothermal amplification. Enzyme inactivation was achieved by heating at 65 °C for 10 min, and reactions were kept at 4 °C until library preparation. No-template (no-DNA) sWGA reactions served as negative controls to check for contamination.

Amplified products were prepared for sequencing using the Native Barcoding Kit 24 V14 (SQK-NBD114.24; Oxford Nanopore Technologies, Oxford, UK) following the manufacturer’s protocol. Barcoded libraries were sequenced on a GridION platform equipped with an R10.4.1 flow cell (FLO-MIN114). Each of the 11 phenotypically negative biopsy samples underwent two independent rounds of selective whole-genome amplification and sequencing.

### 3.5. Natural Transformation Assays for Proof-of-Concept Assessment of Resistance-Associated DNA

To determine whether resistance-associated DNA fragments found in phenotypically *H. pylori*-negative gastric biopsies can induce transformation under laboratory conditions, we conducted in vitro natural transformation assays targeting clarithromycin resistance. The experiments concentrated on the *23S rRNA* A2143G mutation, a well-known factor that can cause high-level macrolide resistance and is directly relevant clinically [3,30,34]. All transformation experiments were carried out in duplicate.

Natural transformation was conducted using the clarithromycin-susceptible reference strain *H. pylori* 26695, following a protocol adapted from Sken et al. [35]. Baseline susceptibility was confirmed with an E-test (MIC = 0.125 µg/mL). Donor *23S rRNA* amplicons carrying the A2143G mutation were generated from gastric biopsy DNA of two dyspeptic patients: one phenotypically *H. pylori*-positive (CKIN12) and one phenotypically negative (CKIN7). Bacterial suspensions were prepared from 48 h cultures of strain 26695 and adjusted to an OD590 of 0.1 in Brucella broth supplemented with 10% fetal bovine serum. After a 6 h pre-incubation under microaerophilic conditions, 100 ng of donor DNA was added to 1 mL of exponentially growing *H. pylori* 26695, and the mixture was incubated under microaerophilic conditions for 18 h. Cultures reached OD590 values ranging from 0.97 to 1.42 across duplicate experiments.

After incubation, 100 µL of each culture was plated in duplicate onto selective agar containing clarithromycin at concentrations ranging from 0.25 to 4 µg/mL, as well as onto antibiotic-free control plates. Negative controls included the untransformed *H. pylori* 26695 recipient strain and a transformation control using a wild-type *23S rRNA* amplicon generated from strain 26695 (Hp26695_23SrRNA). A no-DNA (mock) transformation control and a sterility control consisting of culture medium only were also included (Supplementary Figure S2). Transformation efficiency (TE) was calculated as the mean number of clarithromycin-resistant transformant colonies recovered after selective culture, normalized to the amount of donor DNA used in the transformation assay, and expressed as colony-forming units per microgram of DNA (CFU/µg DNA), according to the following formula:

$$TE=\frac{\mathrm{Mean}\;\mathrm{number}\;\mathrm{of}\;\mathrm{transformants}}{\mathrm{Donor}\;\mathrm{DNA}\;(\mu g)}$$

Colony counts were adjusted for the plated fraction prior to TE estimation.

For each transformation condition, eight colonies per replicate obtained from plates containing 4 µg/mL clarithromycin were randomly selected, subcultured on antibiotic-free medium, and expanded into clonal lines. Whole-genome sequencing (WGS) was performed to confirm the acquisition of the *23S rRNA* A2143G mutation and to identify additional genomic changes in the transformants, as previously described [36,37].

### 3.6. Bioinformatics

#### 3.6.1. Analysis of Targeted ARGs Amplicon Sequences

Raw nanopore reads were adapter-trimmed using Porechop v0.2.4 [38], and read quality was assessed with NanoPlot v1.42.0 [39]. Reads with an average Phred quality score < 10 were removed using NanoFilt v2.8.0 [40]. Additional length-based filtering was applied to retain reads within the expected size range for each amplicon. Filtered reads were aligned to the corresponding reference locus sequences (FASTA) using Minimap2 v2.17 [41]. Alignment files were sorted and indexed with SAMtools v1.20 [42]. Locus-specific read depth (coverage) was estimated from region-restricted alignments using SAMtools, and amplicons with coverage ≤ 2 reads were considered absent.

Consensus amplicon sequences were generated by polishing aligned reads with Racon v1.5.0 [43]. Variant calling was performed with Snippy v4.6.0 [44] by aligning consensus amplicon sequences to the corresponding reference genes from *H. pylori* strain 26695. Identified variants were annotated, and mutations previously reported or predicted to confer antimicrobial resistance were documented for downstream allelic analyses.

#### 3.6.2. Analysis of sWGA Sequences

Raw nanopore reads were adapter-trimmed with Porechop v0.2.4 [38] and quality-filtered (minimum Phred score ≥ 10) using NanoFilt v2.8.0 [40]. Read quality before and after filtering was checked with NanoPlot v1.42.0 [39]. Filtered reads aligned to the *H. pylori* 26695 reference genome and the human reference genome assembly GRCh38.p14 (NCBI RefSeq accession: GCF_000001405.40) using Minimap2 v2.17 [41]. Reads mapping to the human genome were removed with SAMtools v1.20 [42], and *H. pylori*-mapped reads were saved for downstream analyses. These reads were converted to FASTA format and analyzed using DFAST for basic sequence stats, taxonomic assignment, and QC metrics (including completeness and contamination estimates), noting these metrics are based on read fragments rather than non-redundant genome assemblies.

Gene content profiling was conducted using ABRicate v1.0.1 [45] against the Virulence Factor Database (VFDB; release August 2025) and a customized *H. pylori* AMR-associated gene database. Hits meeting thresholds of ≥40% sequence coverage and ≥80% nucleotide identity were retained for virulence- and resistance-associated gene recovery analyses. Results were interpreted as gene recovery rather than definitive gene presence or absence.

#### 3.6.3. Analysis of WGS of Bacterial Transformants

WGS reads from the natural transformation experiments were processed using Porechop [38] for adapter trimming, NanoPlot [39] for QC, and NanoFilt [40] for quality filtering (Q ≥ 10). De novo assembly was performed with Trycycler v0.5.5 [46], combining assemblies generated by Raven, Flye, and Miniasm. Datasets with insufficient read depth to meet Trycycler clustering requirements were excluded from the hybrid consensus assembly step. Assemblies were annotated using Prokka v1.14.6 [47]. The *23S rRNA* gene was extracted through a BLAST-based search using the *H. pylori* 26695 *23S rRNA* sequence as a reference. *23S rRNA* sequences from transformants and the parental strain were aligned to the *H. pylori* 26695 reference using UGENE v52.1 [48] to verify the acquisition of the A2143G macrolide resistance mutation.

#### 3.6.4. Statistical Analysis

All statistical analyses and data visualizations were conducted using R software (version 4.3.2; R Foundation for Statistical Computing, Vienna, Austria). Categorical variables were summarized with frequencies and proportions and compared using the chi-square test or Fisher’s exact test, as appropriate. Continuous variables were expressed as mean ± standard deviation (SD). Normality of continuous variables was evaluated with the Shapiro–Wilk test. For each gene, when normality was confirmed in both groups (Shapiro–Wilk *p* ≥ 0.05), mean values were compared using Welch’s *t*-test to account for unequal variances. All tests were two-tailed, and statistical significance was set at *p* < 0.05.

## 4. Results

### 4.1. Sample Characterization

Samples were obtained from a cross-sectional survey of 220 Congolese patients presenting with dyspepsia. Conventional diagnostic methods included rapid urease test, culture, histology, and immunohistochemistry, although their availability varied across the cohort (Supplementary Table S1). For the molecular analyses presented here, we selected 46 gastric biopsy specimens—23 from phenotypically negative patients and 23 from phenotypically positive patients, as shown in Figure 1. In this subset, phenotypically negative samples were defined as biopsies that underwent at least three of the four diagnostic tests (rapid urease test, culture, histology, and immunohistochemistry) and tested negative in all performed assays. Phenotypically positive samples were identified by positivity in at least one conventional diagnostic test (Supplementary Table S2). This balanced selection aimed to enable comparative analysis while considering differences in bacterial DNA load and detection sensitivity between the phenotypic groups.

### 4.2. Detection and Read Depth of ARG-Associated Loci in Phenotypically Negative Biopsies

**Figure 2:**
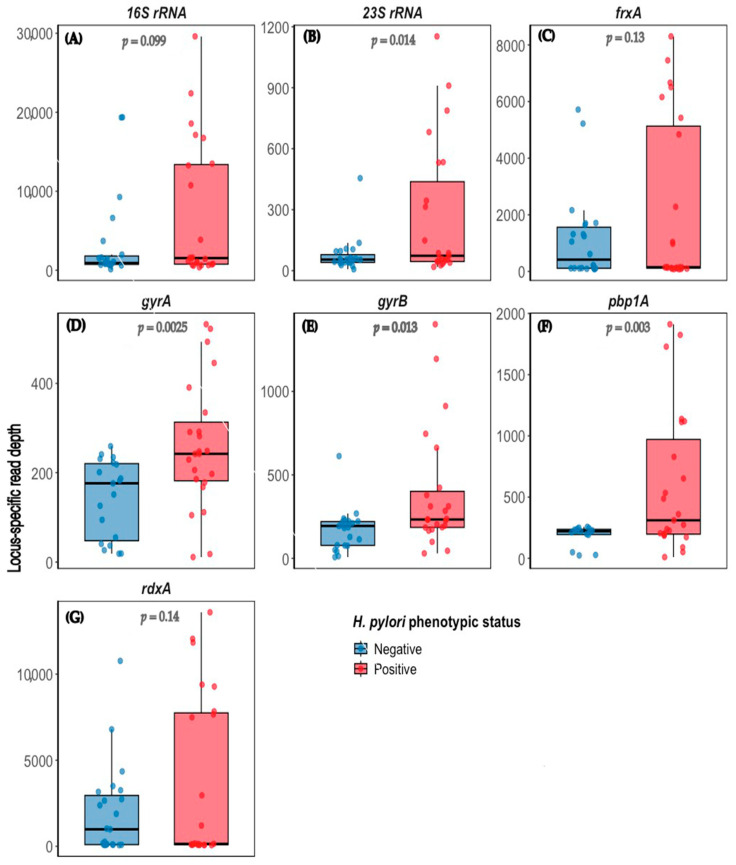
Locus-specific read depth of ARG-associated sequences in gastric biopsies based on *H. pylori* phenotypic status (*n* = 46). Panels (**A**–**G**) display sequencing depth distributions for *16S rRNA* (**A**), *23S rRNA* (**B**), *frxA* (**C**), *gyrA* (**D**), *gyrB* (**E**), *pbp1A* (**F**), and *rdxA* (**G**) loci from biopsies of phenotypically *H. pylori*-positive and -negative dyspeptic patients. Individual points represent samples; boxplots show the median and interquartile range. Group comparisons used Welch’s *t*-test for normally distributed data and the Wilcoxon rank-sum test when normality assumptions were not met. Statistically significant differences are indicated by *p*-values above each panel.

Targeted amplification and long-read sequencing of *H. pylori* AMR-associated loci were performed on all 46 gastric biopsies (Table 1). All seven loci were detectable in nearly all phenotypically positive samples. In phenotypically negative biopsies, detection frequencies remained high for most targets, although lower detection rates were observed for *pbp1A* (65.2%) and, to a lesser extent, *gyrA* (82.6%) and *frxA* (95.7%). Additionally, locus-specific read depth (Figure 2) was higher in phenotypically positive samples compared to phenotypically negative ones. Significant differences were observed for *23S rRNA* (266 ± 334 vs. 76 ± 88.1; *p* = 0.014), *gyrA* (260 ± 144 vs. 143 ± 86.9; *p* = 0.003), *gyrB* (384 ± 360 vs. 172 ± 124; *p* = 0.013), and *pbp1A* (602 ± 590 vs. 188 ± 82.2; *p* = 0.003), with non-significant trends toward higher read depth also noted for *16S rRNA*, *frxA*, and *rdxA*.

Overall, phenotypically positive samples consistently showed a higher recovered sequencing signal across AMR-associated loci. Under standardized amplification and sequencing conditions, these differences reflect variation in *H. pylori* DNA signal between groups rather than qualitative differences in gene content. However, because read depth can be affected by amplification and sequencing biases—including locus-specific amplification efficiency—results are interpreted as relative differences in recovered sequencing signal rather than absolute gene abundance (Supplementary Figure S1). Per-biopsy concordance between phenotypic classification and locus-specific molecular detection (including read depth and key variants) is summarized in Supplementary Table S3. Collectively, these data demonstrate that AMR-associated *H. pylori* loci remain broadly detectable by targeted sequencing in both phenotypic groups, although reduced DNA signal in phenotypically negative biopsies likely contributes to modest decreases in detection at specific loci.

### 4.3. H. pylori Resistance-Associated Mutations by Phenotypic Status

Analysis of AMR-associated genotypes within the targeted *H. pylori* loci revealed a broad distribution of resistance-related mutations in both phenotypically positive and negative biopsies (Table 2). Tetracycline-associated *16S rRNA* mutations (A926T and insG927GC) were detected at low frequencies (<10%) in both groups. In contrast, the *23S rRNA* A2143G mutation—well known as a marker of clarithromycin resistance and treatment failure—was highly prevalent, occurring in nearly half of the samples regardless of phenotypic status (*p* = 0.768). Putative amoxicillin resistance-related variants in *pbp1A*, including substitutions in penicillin-binding domains (S402G, E406A, N562Y, I563T/I/V) and the C-terminal region (S589G, T593A, G595_V596delinsSI, G595_V596insG, V596delinsGGI), were found at variable frequencies without a clear link to phenotype (*p* > 0.05). Mutations in the quinolone resistance-determining region were detected more often in *gyrA* (N87I/K/T, A92T) than in *gyrB* (V437L), but these alterations also showed no association with diagnostic phenotype. Frameshift or disruptive mutations in *frxA* and *rdxA* appeared sporadically (<15%) in both groups. Among all mutations, only the *rdxA* R90K substitution was significantly more common in phenotypically positive biopsies (100% vs. 56.5%; *p* = 0.001), while other mutations showed no meaningful link to phenotypic status. Overall, these results demonstrate that AMR-related mutations are present across samples and reveal heterogeneity in *H. pylori* genotypic profiles, including in phenotypically negative biopsies. Moreover, several well-known resistance mutations—*23S rRNA* A2142G/C, *gyrA* D91N/G/Y, and canonical tetracycline resistance variants in the 926–928 region (AGA926–928TTC, AG926–927GT, A926G, A928C)—were examined but not found in this dataset.

### 4.4. Partial Recovery of H. pylori Sequence Fragments by sWGA in Phenotypically Negative Samples

Selective whole-genome amplification (sWGA), followed by long-read sequencing, enabled partial recovery of *H. pylori*-mapped sequence fragments from gastric biopsies obtained from 11 phenotypically negative patients. Per-sample sequencing yield varied significantly, with total reads ranging from approximately 10,000 to over 230,000 and total bases from about 3.1 × 10^7^ to 7.8 × 10^8^ bp after two sWGA runs. In all samples, one run contributed most of the reads, while the second run consistently provided additional depth. Despite high overall sequencing output, only a small fraction of reads mapped to *H. pylori* (200–440 reads per sample; ~6.1 × 10^5^ to 1.3 × 10^6^ bp), which represented a low percentage of total yield (Supplementary Table S4). This pattern corresponds to low-biomass, host-dominated samples. Variability in *H. pylori*-mapped reads and bases across samples indicates differences in recovered sequencing signals, likely reflecting variation in bacterial DNA availability and sWGA amplification efficiency. Because sWGA can cause uneven amplification and preferential enrichment of certain regions, recovery was partial and heterogeneous across samples. The absence of specific regions does not necessarily indicate the true absence of bacterial genetic material. Importantly, the number of *H. pylori*-mapped bases per sample generally aligned with the total length of recovered *H. pylori* sequence fragments reported in downstream analyses, supporting the internal consistency of the mapping and reporting workflow.

Subsequently, *H. pylori*-mapped reads were converted to FASTA format and analyzed using DFAST to obtain basic sequence statistics and taxonomic assignment. Despite variability in mapped read yield across samples, the GC content remained stable (39.2–44.4%), and estimated contamination levels were low (<12%). Completeness estimates were limited (11–24%), consistent with partial and heterogeneous recovery expected from sWGA-enriched, read-derived fragments rather than non-redundant genome assemblies. Taxonomic assignment to *H. pylori* was consistently supported by GC content, low contamination estimates, and ANI values greater than 91% (Supplementary Table S5).

Gene content analysis based on sWGA-derived *H. pylori*-mapped reads showed recovery of sequence fragments matching antimicrobial resistance-associated genes (ARGs) and virulence-related genes (VRGs) in phenotypically negative patients (Table 3 and Table 4). Because sequence recovery was incomplete and varied across samples, results were interpreted based on gene recovery rather than definitive presence or absence. Among samples, ARG-related loci were recovered in some read-derived fragment sets, with recovery frequencies differing by gene, reflecting uneven coverage and locus-specific recovery patterns. Similarly, virulome profiling revealed partial recovery of well-known functional categories, including motility, immune evasion, acid adaptation, and parts of the *cag* pathogenicity island and secretion systems. Overall, the variation in gene recovery among samples likely results from differences in sequencing signals and fragment completeness rather than true biological absence, suggesting that sWGA can support fragment-based profiling of recovered *H. pylori* genetic features from phenotypically negative biopsies.

Gene recovery was assessed from sWGA-derived *H. pylori*-mapped reads obtained from phenotypically negative gastric biopsy samples. Because sequence recovery was incomplete and variable across samples, results are reported as gene recovery (recovered/not recovered) rather than definitive biological presence or absence. “Samples with gene recovered” indicates the number of samples in which a given antimicrobial resistance gene (ARG) or virulence-related gene (VRG) was detected above the predefined thresholds (≥40% sequence coverage and ≥80% identity). “Mean coverage (%)” represents the average fraction of the gene length covered by matching sequence, calculated only across samples in which the gene was recovered. Variability in gene recovery across samples is expected in low-biomass, sWGA-enriched specimens and may reflect differences in recovered sequencing signal and fragment completeness rather than true gene absence.

These findings support the presence of recoverable *H. pylori* genetic material, including ARG- and VRG-matching fragments, in some phenotypically negative samples, while highlighting the limitations caused by incomplete and variable fragment recovery.

### 4.5. Proof-of-Concept for Natural Transformation Using Biopsy-Derived 23S rRNA Fragments

Natural transformation assays were performed as a proof-of-concept laboratory method to assess whether targeted resistance-associated *H. pylori* DNA fragments recovered from gastric biopsy DNA retained enough sequence integrity to enable homologous recombination under controlled in vitro conditions. We amplified approximately a 2738-base pair *23S rRNA* fragment from biopsy DNA of one phenotypically positive patient (CKIN12_23SrRNA) and one phenotypically negative patient (CKIN7_23SrRNA), and used these products as donor DNA for natural transformation-mediated homologous recombination (allelic exchange) in the clarithromycin-susceptible recipient strain 26695 (Figure 1). Both donor *23S rRNA* fragments contained the A2143G mutation. For negative controls, we used a wild-type *23S rRNA* amplicon amplified from the recipient strain (Hp26695_23SrRNA) and a no-DNA (mock) transformation control (Supplementary Figure S2).

After natural transformation with CKIN12_pos_23SrRNA and CKIN7_neg_23SrRNA PCR products, colonies resistant to clarithromycin were obtained on selective plates containing at least 0.25 µg/mL of clarithromycin, three days after incubation. The average transformation efficiency (TE), calculated from two independent experiments, was approximately 1.6 × 10^3^ CFU/µg and 0.05 × 10^3^ transformants/µg of DNA, respectively. In contrast, no clarithromycin-resistant colonies were observed after transformation with the wild-type amplicon from 26695 or distilled water instead.

**Figure 3:**
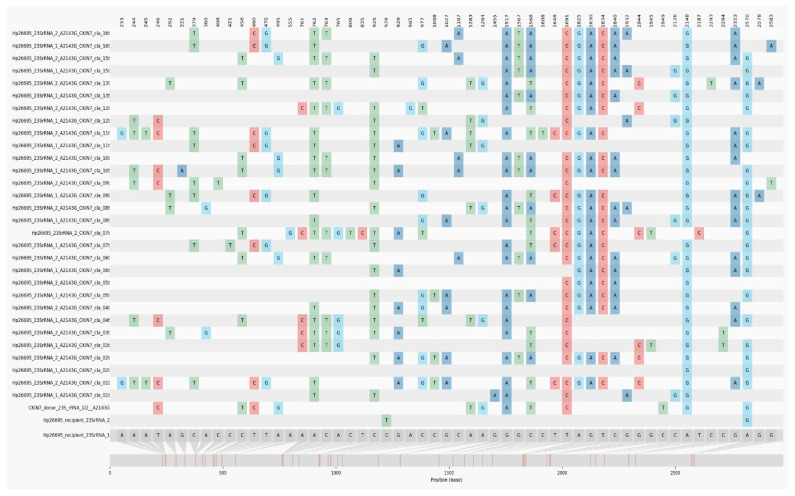
Mapping of *23S rRNA SNPs* in *Helicobacter pylori* recipient, donor (CKIN7), and transformant strains. This figure shows the single-nucleotide polymorphism (SNP) mapping of *23S rRNA* genes in the *H. pylori* 26695 recipient strain, the donor CKIN7 sample (which is phenotypically *H. pylori*-negative), and 15 independent transformants, visualized using *Snipit* from multiple-sequence alignment data. The SNP coordinates are shifted by +5 bp from their usual annotation due to alignment offsets caused by insertions/deletions (indels) at positions 241, 764, and 1943. As a result, the canonical clarithromycin resistance mutation, traditionally annotated as A2143G, is displayed here as A2148G. The dataset includes both *23S rRNA* gene copies from the 26695-recipient strain obtained via whole-genome sequencing (WGS), a consensus *23S rRNA* sequence from the donor biopsy generated by targeted PCR sequencing, and both *23S rRNA* gene copies from the Hp26695 transformants obtained through WGS after selection with 4 µg/mL clarithromycin. Strain naming follows the format: [Background strain]-[targeted gene][mutation]-[donor sample]-[selection condition]-[Clone#]. Notably, natural transformation resulted in allelic exchange, introducing the A2143G mutation (shown as A2148G) into both *23S rRNA* gene copies in all transformants except 07t, where the mutation was detected in only one copy. Additional *23S rRNA* SNPs observed in some transformants are consistent with homologous recombination during natural transformation, but none have been previously linked to clarithromycin resistance.

We further examined a set of 16 independent transformant isolates. The transformant colonies were consistently selected from plates containing 4 µg/mL of clarithromycin (4 µg/mL) (Table 5). Targeted sequencing of the *23S rRNA* gene in the transformants confirmed the presence of resistance-related mutations. All isolates harbored the expected A2143G substitution, regardless of the donor sample phenotype (Figure 3 and Figure S3).

## 5. Discussion

This study demonstrates that *Helicobacter pylori* DNA fragments, including AMR-associated loci, can be recovered from the gastric mucosa of patients who are phenotypically classified as negative by conventional diagnostic methods. By combining targeted long-read amplicon sequencing, sWGA-based enrichment with long-read sequencing, and a proof-of-concept natural transformation assay, we show that phenotypic negativity does not necessarily mean the absence of *H. pylori* genetic material. Instead, a subset of phenotypically negative biopsies contained low-abundance and/or fragmentary *H. pylori* DNA, which has implications for interpreting molecular AMR signals and designing surveillance strategies in low-biomass environments.

Sequencing of targeted AMR-associated loci (*16S rRNA*, *23S rRNA*, *gyrA*, *gyrB*, *rdxA*, *frxA*, and *pbp1A*) revealed that *H. pylori*-derived sequence fragments can be recovered from gastric biopsies of both phenotypically positive and negative individuals, consistent with previous molecular studies [17,21,49]. However, read depth (i.e., recovered reads per locus) was consistently lower in phenotypically negative specimens, aligning with an earlier report [25], indicating a quantitative reduction in *H. pylori* DNA signal rather than a clear qualitative difference. Notably, the non-detection of some loci—particularly single-copy genes (e.g., *gyrA*, *gyrB*, and *pbp1A*)—occurred mainly in low-signal samples and should be interpreted carefully. In this context, failure to detect a specific locus may be due to random sampling at low DNA levels, DNA fragmentation, or primer mismatch, rather than actual absence. In support of this, in silico primer coverage analysis showed variability in predicted amplification efficiency depending on the locus. Overall, these findings suggest that many phenotypically negative biopsies contain *low amounts of H. pylori* DNA and/or DNA in fragmented form, often below the detection limit of phenotypic diagnostic methods, and that molecular results from such low-biomass samples should be interpreted cautiously.

Resistance-associated mutations were identified in both phenotypic groups, including key markers linked to resistance to clarithromycin, fluoroquinolones, and other first-line treatments. However, due to variations in sequencing depth and locus recovery, comparing mutation frequencies between groups should be approached cautiously. Detected resistance-related variants in phenotypically negative samples indicate that AMR-associated *H. pylori* genetic material can be recovered even when phenotypic tests show negative results. Similar observations have been made in other bacteria. In *Mycobacterium tuberculosis*, resistance-conferring mutations can remain detectable by molecular assays after culture conversion and treatment, reflecting the persistence of unculturable bacilli and/or residual DNA [50,51]. In *Streptococcus pneumoniae* and *Neisseria gonorrhoeae*, resistance determinants have been found in molecularly positive but culture-negative samples following antimicrobial treatment [52,53]. Additionally, extracellular DNA with resistance determinants can persist in mucosal environments and be detected by molecular methods even in the absence of recoverable live organisms [54]. In this study, our findings may represent residual *H. pylori* DNA, non-replicating or non-culturable bacteria, or extracellular DNA in the gastric environment [55]. This study was not designed to determine whether these molecular signals contribute to treatment failure or clinically relevant resistance, and since our data do not distinguish among these possibilities, they should not be considered proof of active infection and should be interpreted as a recoverable sequence signal under the applied experimental conditions.

Additionally, changes in the gastric microbial environment may potentially affect *H. pylori* detectability by altering bacterial density, spatial distribution, metabolic activity, or cultivability within the gastric mucosa. Previous studies have indicated that gastric microbiome composition and local inflammatory conditions can influence *H. pylori* persistence and colonization patterns, especially after antimicrobial treatment or in low-biomass conditions [56,57]. Although microbiome profiling was beyond the scope of this study, such host–microbial interactions could contribute to the decreased sensitivity of traditional phenotypic diagnostic methods in some patients.

Advances in molecular and sequencing-based methods have significantly enhanced the characterization of *H. pylori* antimicrobial resistance and host–pathogen interactions beyond the limits of traditional culture-dependent diagnostics. Genomic analyses allow for the detection of resistance-related mutations directly from clinical samples, while broader transcriptomic and proteomic studies have revealed adaptive responses tied to antimicrobial stress, persistence, and bacterial survival strategies. However, most of these methods still depend mainly on culture-positive isolates or samples with high biomass [17,21,49,58], which results in phenotypically negative or low-signal gastric biopsies being underrepresented in routine AMR surveillance. In this context, the current study specifically targeted this diagnostic gap by combining targeted long-read sequencing, sWGA-based enrichment, and proof-of-concept transformation assays to determine if phenotypically negative biopsies contain recoverable *H. pylori*-associated molecular signals. Our results further highlight that interpreting such low-abundance and fragmented DNA signals demands caution, especially considering the risks of contamination, uneven amplification, and uncertainties about bacterial viability or clinical significance.

To better characterize *H. pylori* sequence data in these low-biomass samples, we used sWGA to enrich *H. pylori* DNA from phenotypically negative biopsies. sWGA enabled recovery of *H. pylori*-mapped sequences from these samples, but the data were consistently fragmented and incomplete, likely due to the limited quality and amount of input DNA. As expected, amplification bias and uneven coverage were observed—common issues with sWGA, especially in low-biomass scenario [59,60,61]. Therefore, gene-level analyses relied on gene recovery (i.e., detection depending on sequencing yield and fragment recovery) rather than confirming biological presence or absence. The detection of multiple genomic regions, including AMR-related loci and specific virulence genes, supports the presence of recoverable *H. pylori* DNA fragments and is further validated by consistent quality metrics (such as GC content, ANI values, and contamination estimates). However, incomplete and variable fragment recovery limits broader conclusions, and failure to recover certain loci should not be taken as proof of their true absence.

The natural transformation assay demonstrated that resistance-associated DNA fragments recovered from gastric biopsies maintained enough sequence integrity to mediate homologous recombination in laboratory conditions (in vitro). Donor DNA containing the *23S rRNA* A2143G mutation successfully transformed a susceptible *H. pylori* strain, producing clarithromycin-resistant colonies. This mutation was chosen for transformation experiments as a representative marker of clinically relevant clarithromycin resistance due to its well-documented association with phenotypic resistance and treatment failure, as well as its common presence in *H. pylori* populations from the DRC and other high-prevalence areas [26,62]. Transformation was achieved using DNA from both phenotypically positive and negative samples, although efficiency was lower with the negative samples, likely due to reduced effective template input. While these findings align with the known natural competence of *H. pylori* [9], they should be viewed strictly as experimental results obtained under controlled laboratory conditions and not as proof of in vivo resistance transfer. The frequency, efficiency, and clinical importance of such events in vivo remain unknown.

Several limitations should be considered when interpreting these findings. First, molecular detection of *H. pylori*-associated DNA cannot distinguish between viable bacteria, non-viable organisms, fragmented DNA, or extracellular bacterial material. Therefore, it cannot confirm active infection, persistent colonization, or clinically relevant antimicrobial resistance. This study was designed to examine the recoverability of *H. pylori*-associated molecular signals from phenotypically negative gastric biopsies, rather than to demonstrate active antimicrobial-resistant infection or in vivo transmission potential.

Second, although phenotypically negative biopsies consistently showed lower sequencing read depth and fragmented sequence recovery compared to phenotypically positive biopsies, these samples should be understood as relatively low-biomass specimens rather than definitively *H. pylori*-free gastric tissue. A reduced bacterial load may explain the limited sensitivity of traditional phenotypic diagnostic methods, such as culture, rapid urease testing, histology, and immunohistochemistry. However, the biological importance of low-abundance molecular signals found in these samples remains unclear. Residual DNA from previously damaged, partially cleared, or non-culturable bacterial populations could also contribute to detectable sequence recovery.

Third, despite applying multiple contamination-control measures such as aseptic sample collection and handling, extraction blank controls, purification controls, and PCR no-template controls, low-biomass molecular workflows remain inherently susceptible to low-level contamination, random amplification effects, and amplification bias. No detectable DNA signal or amplification products were observed in the monitored upstream negative controls under the experimental conditions used; however, patient-specific environmental blank controls were not collected during endoscopy, and negative controls were not included during library preparation or sequencing. Therefore, the possibility of low-level contamination introduced during downstream processing cannot be completely ruled out [18,19]. Additionally, a conservative detection threshold was used to limit the interpretation of isolated spurious reads; however, stochastic low-level detection effects cannot be entirely prevented. Furthermore, targeted PCR amplification was not performed systematically in technical replicates for all samples. In low-biomass specimens, random variation in amplification efficiency and limited template availability may lead to occasional false negatives or inconsistent locus recovery. As a result, the absence of amplification, particularly in phenotypically negative biopsies, should be interpreted cautiously. Although two independent sWGA sequencing runs were conducted for some samples, these experiments do not fully replace replicate validation at the targeted PCR level.

Fourth, selective whole-genome amplification (sWGA) inherently results in uneven genome representation and preferential enrichment of certain regions, especially in low-biomass samples with limited pathogen DNA. Consequently, the *H. pylori*-mapped reads recovered from phenotypically negative biopsies were fragmented and incomplete, limiting genome-wide analysis and precluding conclusions about intact genomes, metabolic activity, persistence, or bacterial viability. Therefore, the sequence recovery patterns observed should be interpreted cautiously as partial molecular recovery rather than evidence of complete or biologically intact genomes.

Fifth, the study did not use independent quantitative validation methods, such as quantitative PCR or direct measurement of bacterial load. Therefore, sequencing read depth should not be considered a straightforward measure of bacterial abundance, especially given the potential effects of amplification efficiency and sequencing bias.

Finally, although exclusion criteria included recent antibiotic or bismuth use within 4 weeks and proton pump inhibitor use within 2 weeks before endoscopy, relevant clinical data, such as prior antimicrobial exposure and eradication history, relied partly on patient self-report and may be prone to recall bias. Consequently, we cannot fully exclude the possibility that prior or partial treatment contributed to phenotypic negativity in some cases. Additionally, unmeasured host-related factors, including gastric inflammation, epithelial turnover, and microenvironmental variability, may influence the persistence and detection of low-abundance *H. pylori*-associated DNA within the gastric mucosa [56,57].

Together, these findings indicate that molecular signals linked to *H. pylori* can be detected from phenotypically negative gastric biopsies in low-biomass conditions. However, because detecting low-abundance molecules might indicate residual, fragmented, or non-viable bacterial material, careful interpretation remains essential. Future research using quantitative methods and viability-based techniques will be crucial to understand the biological and clinical significance of these results.

## 6. Conclusions

This study shows that gastric biopsies classified as phenotypically *Helicobacter pylori*-negative can still contain recoverable, low-abundance, and fragmentary *H. pylori*-associated DNA, including loci related to antimicrobial resistance (AMR) and selected virulence-associated sequence fragments. Targeted long-read sequencing and selective whole-genome amplification enabled recovery of molecular signals inaccessible to traditional phenotypic methods, although sequencing depth was consistently lower in phenotypically negative samples, supporting a lower *H. pylori* DNA load. Under controlled laboratory conditions, selected *23S rRNA* resistance-associated fragments retained sufficient sequence integrity to mediate allelic exchange in vitro; however, these results should be viewed as a proof-of-concept observation rather than proof of bacterial viability, active infection, or in vivo resistance transfer.

Overall, these findings highlight that molecular detection in low-biomass gastric samples should be interpreted carefully. Detecting resistance-associated DNA does not necessarily mean there is clinically relevant antimicrobial resistance or biologically significant persistence. Instead, this work underscores important methodological considerations for molecular AMR surveillance and provides an integrated framework for analyzing low-abundance molecular signals related to *H. pylori* in diagnostically challenging clinical samples.

## Figures and Tables

**Table 1:** Detection frequency of ARG-associated loci by targeted sequencing based on *H. pylori* phenotypic status.

ARG Target	*H. pylori* Status of Patients	
	Phenotypically Positive (*N* = 23)	Phenotypically Negative (*N* = 23)
	*n* (%)	*n* (%)
*16S rRNA*	23 (100)	23 (100)
*23S rRNA*	23 (100)	23 (100)
*pbp1A*	23 (100)	15 (65.2)
*gyrA*	23 (100)	19 (82.6)
*gyrB*	23 (100)	23 (100)
*frxA*	23 (100)	22 (95.7)
*rdxA*	23 (100)	23 (100)

**Table 2:** AMR-related mutations in targeted loci based on patient *H. pylori* phenotypic status.

AMR-Related Genotypes (Antibiotics)	*H. pylori* Status of the Patient						*p*-Value
	Phenotypically Positive			Phenotypically Negative			
	*N*	*n*	%	*N*	*n*	%	
*16S rRNA* (tetracycline)							
A926T	23	1	4.35	23	0	0.00	1
insG927GC	23	0	0.00	23	2	8.70	0.489
AGA926–928TTC	23	0	0	23	0	0	
AG926–927GT	23	0	0	23	0	0	
A926G	23	0	0	23	0	0	
A928C	23	0	0	23	0	0	
*23S rRNA* (clarithromycin)							
A2143G	23	13	56.52	23	11	47.83	0.768
A2142G	23	0	0	23	0	0	
A2142C	23	0	0	23	0	0	
*pbp1A* (Amoxicillin)							
N322_E323insD	23	14	60.87	15	12	80.00	0.294
N322D	23	9	39.13	15	1	6.67	0.056
S402G	23	1	4.35	15	0	0.00	1
E406A	23	23	100.00	15	15	100.00	1
V469M	23	2	8.70	15	0	0.00	0.509
N562Y	23	1	4.35	15	0	0.00	1
I563T	23	1	4.35	15	0	0.00	1
I563V	23	0	0.00	15	1	6.67	0.395
S589G	23	22	95.65	15	13	86.67	0.55
T593A	23	2	8.70	15	1	6.67	1
G595_V596delinsSI	23	1	4.35	15	0	0.00	1
G595_V596insG	23	4	17.39	15	1	6.67	0.63
V596delinsGGI	23	1	4.35	15	0	0.00	1
*gyrA* (Levofloxacin)							
N87I	23	4	17.39	19	1	5.26	0.356
N87K	23	1	4.35	19	0	0.00	1
N87T	23	0	0.00	19	1	5.26	0.452
A92T	23	2	8.70	19	0	0.00	0.492
D91N	23	0	0	19	0	0	
D91G	23	0	0	19	0	0	
D91Y	23	0	0	19	0	0	
*gyrB* (Levofloxacin)							
V437L	23	1	4.35	23	0	0.00	1
*frxA* (Metronidazole)							
A173fs	23	0	0.00	22	1	4.55	0.489
A70fs	23	0	0.00	22	1	4.55	0.489
E57fs	23	1	4.35	22	0	0.00	1
G69fs	23	1	4.35	22	0	0.00	1
M126fs	23	2	8.70	22	0	0.00	0.489
N124fs	23	0	0.00	22	1	4.55	0.489
S130del.disruptive	23	1	4.35	22	0	0.00	1
S130fs	23	3	13.04	22	3	13.64	1
W137 *	23	1	4.35	22	0	0.00	1
*rdxA* (Metronidazole)							
W52 *	23	0	0.00	23	2	8.70	0.489
Q102 *	23	1	4.35	23	0	0.00	1
R90K	23	23	100.00	23	13	56.52	0.001
H97T	23	1	4.35	23	0	0.00	1
P106T	23	0	0.00	23	1	4.35	1
S108A	23	1	4.35	23	0	0.00	1
A118S	23	1	4.35	23	0	0.00	1

Abbreviations: AMR, antimicrobial resistance. *N* = total number of samples analyzed; *n* = number of samples with the indicated mutation. * indicates a premature stop codon (nonsense mutation). fs, frameshift mutation; del, deletion; ins, insertion; delins, deletion–insertion event.

**Table 3:** Recovery and coverage of *Helicobacter pylori ARG* loci from sWGA-derived reads.

ARGs	Samples Analyzed	Samples with Gene Recovered	Mean Coverage (%)	Interpretation
*16S rRNA*	11	4	81.5%	Consistently recovered
*23S rRNA*	11	2	79.2%	Moderately recovered
*ABC_OPPA*	11	1	79.9%	Moderately recovered
*ABC_OPPB*	11	3	58.3%	Moderately recovered
*ABC_OPPC*	11	3	85.5%	Consistently recovered
*ABC_transporter*	11	2	60%	Moderately recovered
*MSF*	11	9	76.7%	Moderately recovered
*OorDABC1*	11	3	84.2%	Consistently recovered
*OorDABC2*	11	6	84.1%	Consistently recovered
*OorDABC3*	11	1	75.1%	Moderately recovered
*OorDABC4*	11	6	73.5%	Moderately recovered
*PorCDBA1*	11	1	98.6%	Consistently recovered
*PorCDBA2*	11	2	84.2%	Consistently recovered
*PorCDBA3*	11	4	92.6%	Consistently recovered
*PorCDBA4*	11	2	81.8%	Consistently recovered
*RND_MFP*	11	3	89.6%	Consistently recovered
*RND_TP*	11	6	86.9%	Consistently recovered
*RodA1*	11	4	75.6%	Moderately recovered
*SecD*	11	4	69.7%	Moderately recovered
*abc_NikA*	11	5	85.5%	Consistently recovered
*bmrA*	11	4	66.5%	Moderately recovered
*codA*	11	1	43.5%	Partial recovery
*copA*	11	3	55.8%	Moderately recovered
*copA2*	11	2	67.3%	Moderately recovered
*dapF*	11	5	74.8%	Moderately recovered
*ddpA*	11	3	75%	Moderately recovered
*ddpB*	11	3	63.5%	Moderately recovered
*fdxA*	11	3	80.5%	Consistently recovered
*fdxB*	11	2	89.8%	Consistently recovered
*feoB*	11	1	88.2%	Consistently recovered
*fldA*	11	4	68.1%	Moderately recovered
*frxA*	11	2	97.2%	Consistently recovered
*ftsI*	11	2	44.4%	Partial recovery
*fur*	11	6	79.4%	Moderately recovered
*fusA*	11	3	77.8%	Moderately recovered
*hcpA*	11	1	86.7%	Consistently recovered
*hefA*	11	2	59.4%	Moderately recovered
*hefB*	11	1	98.4%	Consistently recovered
*hefC*	11	5	63.5%	Moderately recovered
*hefD*	11	1	41.5%	Partial recovery
*hefE*	11	1	70.4%	Moderately recovered
*hefF*	11	2	57.9%	Moderately recovered
*hefG*	11	1	61.7%	Moderately recovered
*hefH*	11	1	74.9%	Moderately recovered
*hefI*	11	3	63.8%	Moderately recovered
*hypothP*	11	4	83.1%	Consistently recovered
*infB*	11	1	55.8%	Moderately recovered
*kefB*	11	3	71.8%	Moderately recovered
*ketoGP*	11	2	81.3%	Consistently recovered
*lepA*	11	5	77.5%	Moderately recovered
*llm*	11	6	67.7%	Moderately recovered
*lytB*	11	3	90.5%	Consistently recovered
*mdaB*	11	3	69.3%	Moderately recovered
*mreB*	11	6	83.5%	Consistently recovered
*mreC*	11	6	84.8%	Consistently recovered
*msbA*	11	1	65.5%	Moderately recovered
*multidrug_transporter*	11	3	81.6%	Consistently recovered
*norM1*	11	2	68.7%	Moderately recovered
*norM2*	11	1	94.2%	Consistently recovered
*omp11*	11	1	99.6%	Consistently recovered
*pbp1A*	11	1	45%	Partial recovery
*pbp2*	11	5	74.1%	Moderately recovered
*pbp4*	11	5	75.9%	Moderately recovered
*poP*	11	1	72.9%	Moderately recovered
*rdxA*	11	4	74.5%	Moderately recovered
*recA*	11	2	63.6%	Moderately recovered
*rfaF*	11	2	68.2%	Moderately recovered
*ribF*	11	2	82.2%	Consistently recovered
*rnc*	11	2	90.2%	Consistently recovered
*rnd_OMP*	11	4	87.3%	Consistently recovered
*rpl22*	11	1	99.2%	Consistently recovered
*rpoB*	11	2	64.1%	Moderately recovered
*rps4*	11	8	81.2%	Consistently recovered
*rpsU*	11	1	44.1%	Partial recovery
*sodB*	11	1	58%	Moderately recovered
*spoT*	11	2	63.6%	Moderately recovered
*tetA*	11	1	84.3%	Consistently recovered
*tufB*	11	1	42.5%	Partial recovery

**Table 4:** Recovery and coverage of VRGs of *H. pylori* loci from sWGA-derived reads.

**VRGs**	**Samples Analyzed**	**Samples** **with** **Gene** **Recovered**	**Mean** **Coverage** **(%)**	**Interpretation**
*HP0256*	11	4	97.1%	Consistently recovered
*babA/hopS*	11	1	61.9%	Moderately recovered
*babB/hopT*	11	2	70.7%	Moderately recovered
*cag1*	11	4	83.4%	Consistently recovered
*cag2*	11	5	60.8%	Moderately recovered
*cag3*	11	2	77.6%	Moderately recovered
*cagA*	11	3	84.1%	Consistently recovered
*cagD*	11	2	97.6%	Consistently recovered
*cagF*	11	3	98.4%	Consistently recovered
*cagG*	11	4	99%	Consistently recovered
*cagH*	11	3	68.3%	Moderately recovered
*cagM*	11	2	46.7%	Partial recovery
*cagP*	11	1	75.7%	Moderately recovered
*cagQ*	11	2	93.6%	Consistently recovered
*cagS*	11	2	76.9%	Moderately recovered
*cagU*	11	1	99.2%	Consistently recovered
*cagZ*	11	1	64.3%	Moderately recovered
*cds6*	11	4	71.2%	Moderately recovered
*cheV1*	11	1	41.7%	Partial recovery
*cheV2*	11	2	51.7%	Moderately recovered
*flaA*	11	2	63.5%	Moderately recovered
*flaB*	11	2	88.1%	Consistently recovered
*flaG*	11	5	87.2%	Consistently recovered
*flgA*	11	4	73.5%	Moderately recovered
*flgB*	11	2	61.2%	Moderately recovered
*flgC*	11	3	79%	Moderately recovered
*flgD*	11	3	70.4%	Moderately recovered
*flgE*	11	1	57.8%	Moderately recovered
*flgE_1*	11	2	47.9%	Partial recovery
*flgG*	11	8	78.6%	Moderately recovered
*flgH*	11	2	87.5%	Consistently recovered
*flgI*	11	2	92.4%	Consistently recovered
*flgK*	11	3	56.3%	Moderately recovered
*flgL*	11	2	54.5%	Moderately recovered
*flgM*	11	1	99.1%	Consistently recovered
*flgR*	11	6	79.3%	Moderately recovered
*flgS*	11	4	83%	Consistently recovered
*flhA*	11	1	66.2%	Moderately recovered
*flhB*	11	3	93.9%	Consistently recovered
*flhB2*	11	3	88.1%	Consistently recovered
*flhF*	11	1	96.5%	Consistently recovered
*fliA*	11	2	87.8%	Consistently recovered
*fliD*	11	3	67.3%	Moderately recovered
*fliE*	11	3	82.3%	Consistently recovered
*fliF*	11	6	59.6%	Moderately recovered
*fliG*	11	5	80.4%	Consistently recovered
*fliH*	11	5	76.5%	Moderately recovered
*fliI*	11	4	64.3%	Moderately recovered
*fliL*	11	2	51.4%	Moderately recovered
*fliM*	11	6	88.3%	Consistently recovered
*fliN*	11	2	89.1%	Consistently recovered
*fliP*	11	3	95.9%	Consistently recovered
*fliQ*	11	4	94.8%	Consistently recovered
*fliR*	11	4	79.3%	Moderately recovered
*fliS*	11	2	84.7%	Consistently recovered
*fliY*	11	7	85.6%	Consistently recovered
*futA*	11	5	75.6%	Moderately recovered
*futB*	11	2	83.2%	Consistently recovered
*futC*	11	1	47.1%	Partial recovery
*gluE*	11	2	90.9%	Consistently recovered
*gluP*	11	2	63.6%	Moderately recovered
*hopH*	11	2	51.2%	Moderately recovered
*hopZ*	11	1	81.1%	Consistently recovered
*kdtB*	11	3	97.8%	Consistently recovered
*lpxB*	11	2	69.1%	Moderately recovered
*motA*	11	1	78%	Moderately recovered
*motB*	11	1	98%	Consistently recovered
*napA*	11	4	88.4%	Consistently recovered
*neuA/flmD*	11	3	70.7%	Moderately recovered
*pdxA*	11	3	75.9%	Moderately recovered
*pdxJ*	11	4	83.1%	Consistently recovered
*pflA*	11	3	51.7%	Moderately recovered
*pseB*	11	7	86.1%	Consistently recovered
*pseC*	11	1	58.7%	Moderately recovered
*rfaC*	11	3	93.5%	Consistently recovered
*rfaJ*	11	8	74.4%	Moderately recovered
*rfbD*	11	4	77.2%	Moderately recovered
*rfbM*	11	7	69.8%	Moderately recovered
*sabA/hopP*	11	1	60.4%	Moderately recovered
*tlpA*	11	3	65.4%	Moderately recovered
*tlpB*	11	2	43.7%	Partial recovery
*tlpC*	11	5	85%	Consistently recovered
*ureA*	11	3	68.7%	Moderately recovered
*ureB*	11	3	63.5%	Moderately recovered
*ureE*	11	3	84.4%	Consistently recovered
*ureF*	11	2	79.8%	Moderately recovered
*ureG*	11	5	87.9%	Consistently recovered
*ureH*	11	5	96.3%	Consistently recovered
*ureI*	11	4	69.9%	Moderately recovered
*vacA*	11	1	41.8%	Partial recovery
*virB1*	11	2	46.5%	Partial recovery
*virB11*	11	2	52.1%	Moderately recovered
*virB2/cagC*	11	3	82.9%	Consistently recovered
*virB4/cagE*	11	2	58.1%	Moderately recovered
*virB5/cagL*	11	1	62.8%	Moderately recovered
*virB6/cagW*	11	1	68.1%	Moderately recovered
*virB7/cagT*	11	1	96.1%	Consistently recovered
*virB8/cagV*	11	1	97.2%	Consistently recovered
*virB9/cagX*	11	1	60.9%	Moderately recovered
*virD4*	11	1	66.8%	Moderately recovered
*wbcJ*	11	2	97.7%	Consistently recovered
*wbpB*	11	4	84.9%	Consistently recovered
*ylxH*	11	3	64.7%	Moderately recovered

**Table 5:** Natural transformation efficiency of *H. pylori* strain 26695 using *23S rRNA* PCR product derived from gastric biopsies. Transformation efficiency was calculated by adjusting colony counts based on the plated fraction of 0.1 mL out of a 1 mL transformation mixture. This adjusted count was then normalized to the amount of purified donor amplicon DNA (1 µg), resulting in transformation efficiency values after selection on clarithromycin-containing plates (4 µg/mL).

Fragment	Mutated Allele Before Transformation	Mutated Allele After Transformation	MIC (µg/mL)	Transformation Efficiency CFU/µg
CKIN12_pos_23SrRNA	A2143G	A2143G	>4	1.6 × 10^3^
CKIN7_neg_23SrRNA	A2143G	A2143G	>4	0.05 × 10^3^
Hp26695strain_23SrRNA	WT	WT	0.125	0
Distilled water	No DNA	WT	0.125	0

## Data Availability

All nucleotide sequence data generated in this study have been submitted to the National Center for Biotechnology Information (NCBI). Draft genome assemblies for two transformant strains have been assigned accession numbers: *Hp26695_23SrRNA_A2143G_CKIN12_CLA_t12* (JBVMAB000000000) and *Hp26695_23SrRNA_A2143G_CKIN12_CLA_t15* (JBVMAC000000000). Additional genome submissions are currently being processed. Targeted amplicon sequencing datasets for antimicrobial resistance-related loci have also been submitted. Accession numbers assigned for *16S rRNA* and amplicon *23S rRNA* sequences range from PZ022848 to PZ022893 and PZ022898 to PZ022943, respectively, representing sequences from both phenotypically positive and negative gastric biopsy specimens. Raw Oxford Nanopore sequencing reads from selective whole-genome amplification (sWGA) of gastric biopsy DNA have been deposited in the Sequence Read Archive (SRA) under BioProject PRJNA1426731 and SRA study accession SRP693777. Individual run accessions include SRR38218846 to SRR38218856. Accession numbers for all pending datasets will be provided upon public release. In the meantime, all relevant data supporting the findings of this study are available within the article, Supplementary Materials, or from the corresponding author upon reasonable request.

## Supplementary Materials

The following supporting information can be downloaded at: https://www.mdpi.com/article/10.3390/biom16060765/s1, Supplementary Table S1: Distribution of positive and negative results by *H. pylori* conventional diagnostic method; Supplementary Table S2: Conventional diagnostic results per biopsy; Supplementary Table S3: Concordance between traditional *H. pylori* phenotypic status and molecular detection of AMR-associated targeted loci in 46 gastric biopsies; Supplementary Table S4: Per-sample FASTQ file sequencing yield and *H. pylori* mapping statistics from two combined sWGA sequencing runs; Supplementary Table S5: Recovery metrics of *H. pylori* fragment sequences from *H. pylori* phenotypically negative gastric biopsies using the DFAST platform; Supplementary Table S6: Primers used for PCR amplification of antimicrobial resistance genes in *H. pylori*; Supplementary Table S7: In silico evaluation of primer binding and predicted amplification efficiency for *H. pylori* antimicrobial resistance genes; Supplementary Figure S1: Global ARGs sequence depth in gastric biopsies from phenotypically negative and positive patients; Supplementary Figure S2: Growth dynamics of *H. pylori* during natural transformation and clarithromycin selection (D1–D3); Supplementary Figure S3: Mapping of 23S rRNA SNPs in *H. pylori* recipient, donor (CKIN12), and transformant strains; Supplementary Data File S1: Afro_Hp_ARGs_sequences; Supplementary Data File S2: Afro_ARGs_Primers_Blast_Matches.
