# Supplementary material for: Low-Abundance and Fragmentary Helicobacter pylori DNA Detected in Phenotypically Negative Gastric Biopsies Using Targeted Sequencing
Source: Biomolecules. 2026 May 22;16(6):765. doi: 10.3390/biom16060765 (PMC13296529; doi:10.3390/biom16060765)
Supplement: Supplementary file 1 [file biomolecules-16-00765-s001.zip › Hp_sWGA.Supplementary.2026.05.21YY.revised.pdf]

SUPPLEMENTARY DATA

Supplementary Table S1. Distribution of positive and negative results by *H. pylori* conventional diagnostic method

Diagnostic method	Positive	Negative	NA	Total
	n (%)	*n* (%)	*n* (%)	*N* (%)
Rapid urease test (RUT)	26 (11.8)	29 (13.2)	165 (75.0)	220 (100)
Culture	111 (50.5)	109 (49.5)	0 (0.0)	220 (100)
Histology	101 (45.9)	108 (49.1)	11 (5.0)	220 (100)
Immunohistochemistry (IHC)	102 (50.5)	115 (49.5)	3 (1.4)	220 (100)

Supplementary Table S2. Conventional diagnostic results per biopsy

In this study, "Phenotypically positive" was defined as positivity in at least one conventional diagnostic test (rapid urease test, culture, histology, or immunohistochemistry). "Phenotypically negative" samples were defined as samples that underwent at least three of the four routine diagnostic tests (including rapid urease test, culture, histology, and immunohistochemistry) and tested negative in all performed assays.

Biopsy ID (+ / - / NA)	*H. pylori* phenotype (Pos/Neg)	Rapid urease test (RUT) (+ / - / NA)	Culture (+ / - / NA)	Histology (+ / - / NA)	Immunohistochemistry (IHC) (+ / - / NA)
CKIN1	Pos	-	+	-	-
CKIN10	Pos	+	+	-	-
CKIN12	Pos	+	+	NA	+
CKIN14	Pos	+	+	-	-
CKIN15	Pos	+	+	NA	+
CKIN17	Pos	+	+	NA	+
CKIN22	Pos	-	+	+	+
CKIN24	Pos	+	+	-	+
CKIN25	Pos	+	+	NA	+
CKIN29	Pos	+	+	+	+
CKIN3	Pos	-	+	-	-
CKIN31	Pos	-	+	NA	-
CKIN32	Pos	+	+	+	+
CKIN34	Pos	NA	+	+	+
CKIN35	Pos	NA	+	+	+
CKIN36	Pos	NA	+	+	+
CKIN39	Pos	+	+	-	+
CKIN41	Pos	+	+	+	+
CKIN44	Pos	+	+	+	+
CKIN5	Pos	-	+	-	-
CKIN6	Pos	+	+	NA	+
CKIN8	Pos	+	+	-	-
CKIN9	Pos	+	+	+	+
CKIN11	Neg	-	-	-	-
CKIN13	Neg	-	-	-	-
CKIN16	Neg	-	-	-	-
CKIN18	Neg	-	-	-	-
CKIN19	Neg	-	-	-	-
CKIN20	Neg	-	-	-	-
CKIN21	Neg	-	-	-	-
CKIN23	Neg	-	-	-	-
CKIN30	Neg	-	-	-	-
CKIN33	Neg	NA	-	-	-
CKIN38	Neg	-	-	-	-
CKIN46	Neg	NA	-	-	-
CKIN48	Neg	-	-	-	-

CKIN53	Neg	**-**	**-**	**-**	**-**
CKIN55	Neg	**-**	**-**	**-**	**-**
CKIN61	Neg	**NA**	**-**	**-**	**-**
CKIN62	Neg	**NA**	**-**	**-**	**-**
CKIN63	Neg	**-**	**-**	**NA**	**-**
CKIN64	Neg	**NA**	**-**	**-**	**-**
CKIN67	Neg	**NA**	**-**	**-**	**-**
CKIN7	Neg	**-**	**-**	**NA**	**-**
KIN1	Neg	**NA**	**-**	**-**	**-**
KIN2	Neg	**NA**	**-**	**-**	**-**

Supplementary Table S3. Concordance between traditional *H. pylori* phenotypic status and molecular detection of AMR-associated targeted loci in 46 gastric biopsies.

Biopsy ID	Phenotype	All targeted loci detected (Y/N)	Global targeted-loci read depth (proxy)	*16S rRNA*	*23S rRNA*	*gyrB*	*rdxA*	*pbp1A*	*gyrA*	*frxA*
CKIN1	Pos	Y	2489.67	Y; depth=1165.34; mut=-	Y; depth=73.2; mut=A2143G	Y; depth=311.74; mut=-	Y; depth=133.47; mut=R90K	Y; depth=359.91; mut=S589G	Y; depth=291.07; mut=-	Y; depth=154.94; mut=-
CKIN10	Pos	Y	2308.16	Y; depth=1047.74; mut=-	Y; depth=54.6; mut=A2143G	Y; depth=284.33; mut=-	Y; depth=127.34; mut=R90K	Y; depth=310.3; mut=S589G	Y; depth=334.79; mut=-	Y; depth=149.06; mut=M126fs
CKIN12	Pos	Y	2308.16	Y; depth=1606.47; mut=-	Y; depth=148.6; mut=A2143G	Y; depth=912.35; mut=-	Y; depth=143.8; mut=R90K	Y; depth=1119.21; mut=S589G	Y; depth=522.3; mut=-	Y; depth=144.15; mut=-
CKIN14	Pos	Y	1958.87	Y; depth=927.01; mut=-	Y; depth=54.41; mut=A2143G	Y; depth=232.67; mut=-	Y; depth=98.31; mut=R90K	Y; depth=273.65; mut=S589G	Y; depth=247.55; mut=-	Y; depth=125.27; mut=-
CKIN15	Pos	Y	2307.73	Y; depth=1433.58; mut=-	Y; depth=39.14; mut=A2143G	Y; depth=186.23; mut=-	Y; depth=101.93; mut=R90K	Y; depth=191.47; mut=S589G	Y; depth=229.23; mut=-	Y; depth=126.15; mut=-
CKIN17	Pos	Y	1302.11	Y; depth=576.53; mut=-	Y; depth=26.25; mut=A2143G	Y; depth=184.47; mut=-	Y; depth=75.44; mut=R90K	Y; depth=184.19; mut=S589G	Y; depth=178.09; mut=-	Y; depth=77.14; mut=-
CKIN22	Pos	Y	1414.61	Y; depth=622.81; mut=-	Y; depth=45.06; mut=A2143G	Y; depth=165.38; mut=-	Y; depth=86.11; mut=R90K	Y; depth=204.35; mut=S589G	Y; depth=197.23; mut=-	Y; depth=93.66; mut=-
CKIN24	Pos	Y	1465.96	Y; depth=649.28; mut=-	Y; depth=32.9; mut=A2143G	Y; depth=203.84; mut=-	Y; depth=92.59; mut=R90K	Y; depth=202.75; mut=S589G	Y; depth=185.32; mut=-	Y; depth=99.28; mut=-
CKIN25	Pos	Y	49457.24	Y; depth=29606.51; mut=-	Y; depth=910.36; mut=-	Y; depth=1194.84; mut=-	Y; depth=9392.25; mut=R90K	Y; depth=1911.72; mut=S589G	Y; depth=281.81; mut=-	Y; depth=6159.75; mut=-
CKIN29	Pos	Y	35325.91	Y; depth=13495.48; mut=-	Y; depth=533.62; mut=-	Y; depth=423.28; mut=-	Y; depth=12054.48; mut=R90K	Y; depth=828.89; mut=S589G; T593A	Y; depth=532.54; mut=-	Y; depth=7457.62; mut=-
CKIN3	Pos	Y	846.19	Y; depth=379.25; mut=-	Y; depth=18.46; mut=A2143G	Y; depth=98.69; mut=-	Y; depth=66.99; mut=R90K	Y; depth=89.42; mut=S589G	Y; depth=111.33; mut=-	Y; depth=82.05; mut=S130fs
CKIN31	Pos	Y	27697.98	Y; depth=10752.83; mut=-	Y; depth=314.2; mut=-	Y; depth=195.51; mut=-	Y; depth=7493.98; mut=-	Y; depth=533.91; mut=V469M; G595_V596i	Y; depth=104.73; mut=A92T;N87I	Y; depth=8302.82; mut=E57fs

								nsG; S589G; T593A		
CKIN32	Pos	Y	45553.9	Y; depth=22392.53; mut=-	Y; depth=787.55; mut=-	Y; depth=662.55; mut=-	Y; depth=13592.95; mut=R90K	Y; depth=1113.22; mut=S589G	Y; depth=493.32; mut=-	Y; depth=6511.78; mut=-
CKIN34	Pos	Y	37598.91	Y; depth=16744.11; mut=-	Y; depth=531.54; mut=-	Y; depth=236.31; mut=-	Y; depth=11836.47; mut=R90K	Y; depth=1137.55; mut=S589G	Y; depth=445.75; mut=-	Y; depth=6667.19; mut=W137*
CKIN35	Pos	Y	30767.47	Y; depth=18570.21; mut=-	Y; depth=681.99; mut=-	Y; depth=746.06; mut=-	Y; depth=7660.03; mut=R90K	Y; depth=1824.95; mut=G595_V596insG;	Y; depth=242.16; mut=N87I	Y; depth=1042.07; mut=-
CKIN36	Pos	Y	28770.47	Y; depth=13265.18; mut=-	Y; depth=343.99; mut=-	Y; depth=311.41; mut=V437L	Y; depth=9279.72; mut=R90K	Y; depth=487.22; mut=V596delinsGGI; S589G	Y; depth=242.12; mut=A92T;N87K	Y; depth=4840.84; mut=-
CKIN39	Pos	Y	9197.59	Y; depth=3851.35; mut=-	Y; depth=57.25; mut=-	Y; depth=29.53; mut=-	Y; depth=2957.7; mut=R90K	Y; depth=9.78; mut=S589G	Y; depth=10.87; mut=-	Y; depth=2281.11; mut=-
CKIN41	Pos	Y	34974.99	Y; depth=17135.67; mut=-	Y; depth=1152.64; mut=-	Y; depth=1402.52; mut=-	Y; depth=7838.74; mut=R90K	Y; depth=1728.44; mut=S589G; V469M	Y; depth=291.59; mut=N87I	Y; depth=5425.39; mut=-
CKIN44	Pos	Y	3963.52	Y; depth=1579.94; mut=A926T	Y; depth=86.55; mut=-	Y; depth=45.03; mut=-	Y; depth=1204.01; mut=R90K	Y; depth=50.84; mut=G595_V596insG; N562Y; S589G	Y; depth=17.74; mut=N87I	Y; depth=979.41; mut=G69fs
CKIN5	Pos	Y	1636.81	Y; depth=724.13; mut=-	Y; depth=44.64; mut=A2143G	Y; depth=231.91; mut=-	Y; depth=81.96; mut=R90K	Y; depth=238.31; mut=S589G	Y; depth=205.88; mut=-	Y; depth=109.97; mut=S130fs
CKIN6	Pos	Y	3347.51	Y; depth=1536.25; mut=-	Y; depth=86.26; mut=A2143G	Y; depth=378.96; mut=-	Y; depth=164.72; mut=R90K	Y; depth=651.8; mut=G595_V596insG; I563T; S402G; S589G	Y; depth=390.81; mut=-	Y; depth=138.71; mut=M126fs
CKIN8	Pos	Y	1797.66	Y; depth=809.32; mut=-	Y; depth=62.61; mut=A2143G	Y; depth=225.49; mut=-	Y; depth=94.64; mut=R90K	Y; depth=225.17; mut=S589G	Y; depth=248.71; mut=-	Y; depth=131.73; mut=-
CKIN9	Pos	Y	1403.82	Y; depth=681.96; mut=-	Y; depth=43.42; mut=A2143G	Y; depth=173.31; mut=-	Y; depth=74.11; mut=R90K	Y; depth=172.61; mut=S589G	Y; depth=167.81; mut=-	Y; depth=90.6; mut=S130fs
CKIN11	Neg	Y	2308.16	Y; depth=784.24; mut=-	Y; depth=55.06; mut=A2143G	Y; depth=268.42; mut=-	Y; depth=84.47; mut=R90K	Y; depth=250.69; mut=S589G	Y; depth=234.65; mut=-	Y; depth=101.86; mut=S130fs
CKIN13	Neg	Y	1337.2	Y; depth=607.96; mut=-	Y; depth=40.26; mut=A2143G	Y; depth=196.47; mut=-	Y; depth=66.96; mut=R90K	Y; depth=198.31; mut=S589G	Y; depth=151.24; mut=-	Y; depth=76.01; mut=-
CKIN16	Neg	Y	1489.94	Y; depth=652.77; mut=-	Y; depth=40.25;	Y; depth=210.56; mut=-	Y; depth=84.32;	Y; depth=220.75; mut=S589G	Y; depth=176.2; mut=-	Y; depth=105.09;

					mut=A2143G		mut=R90K			mut=S130fs
CKIN18	Neg	Y	1561.97	Y; depth=734.34; mut=-	Y; depth=36.97; mut=A2143G	Y; depth=201.86; mut=-	Y; depth=83.96; mut=R90K	Y; depth=227.25; mut=S589G	Y; depth=186.88; mut=-	Y; depth=90.71; mut=-
CKIN19	Neg	Y	1773.01	Y; depth=825.97; mut=-	Y; depth=41.71; mut=A2143G	Y; depth=228.34; mut=-	Y; depth=109.41; mut=R90K	Y; depth=233.28; mut=S589G	Y; depth=222.02; mut=-	Y; depth=112.28; mut=-
CKIN20	Neg	Y	1755.32	Y; depth=758.34; mut=-	Y; depth=57.9; mut=A2143G	Y; depth=222.33; mut=-	Y; depth=118.07; mut=R90K	Y; depth=240.7; mut=S589G	Y; depth=241.06; mut=-	Y; depth=116.91; mut=-
CKIN21	Neg	Y	1641.16	Y; depth=711.56; mut=-	Y; depth=46.24; mut=A2143G	Y; depth=222.08; mut=-	Y; depth=92.7; mut=R90K	Y; depth=228.64; mut=S589G	Y; depth=230.85; mut=-	Y; depth=109.09; mut=-
CKIN23	Neg	Y	1562.32	Y; depth=689.47; mut=-	Y; depth=43.44; mut=A2143G	Y; depth=200.63; mut=-	Y; depth=75.22; mut=R90K	Y; depth=217.08; mut=S589G	Y; depth=218.52; mut=-	Y; depth=117.95; mut=S130fs
CKIN30	Neg	Y	17818	Y; depth=1140.62; mut=-	Y; depth=58.87; mut=-	Y; depth=78.43; mut=-	Y; depth=10768.69; mut=R90K	Y; depth=27.43; mut=S589G;	Y; depth=26.33; mut=-	Y; depth=5717.63; mut=-
CKIN33	Neg	N	12159.54	Y; depth=122.29; mut=-	Y; depth=6.86; mut=-	Y; depth=12.63; mut=-	Y; depth=6794.69; mut=-	N; depth=0; mut=-	N; depth=1; mut=-	Y; depth=5223.06; mut=A173fs, A70fs
CKIN38	Neg	Y	12110.98	Y; depth=6618.38; mut=-	Y; depth=23; mut=-	Y; depth=7.37; mut=-	Y; depth=3256.33; mut=-	Y; depth=23.71; mut=T593A	Y; depth=18.83; mut=-	Y; depth=2163.36; mut=N124fs
CKIN46	Neg	Y	26390.54	Y; depth=19341.3; mut=-	Y; depth=455.02; mut=-	Y; depth=612.73; mut=-	Y; depth=4352.68; mut=-	Y; depth=190.56; mut=E406A	Y; depth=125.84; mut=-	Y; depth=1312.4; mut=-
CKIN48	Neg	N	4225.87	Y; depth=1559.28; mut=-	Y; depth=53.98; mut=-	Y; depth=113.61; mut=-	Y; depth=1887.92; mut=-	N; depth=0.37; mut=-	N; depth=0; mut=-	Y; depth=611.09; mut=-
CKIN53	Neg	N	4657.46	Y; depth=1618.7; mut=insG927GC	Y; depth=94.8; mut=-	Y; depth=186.19; mut=-	Y; depth=1027.37; mut=-	N; depth=1; mut=-	Y; depth=18.49; mut=-	Y; depth=1711.92; mut=-
CKIN55	Neg	N	8235.34	Y; depth=3704.74; mut=-	Y; depth=107.23; mut=-	Y; depth=76.41; mut=-	Y; depth=2649.17; mut=-	N; depth=0; mut=-	N; depth=0; mut=-	Y; depth=1697.79; mut=-
CKIN61	Neg	Y	14159.52	Y; depth=9272.46; mut=-	Y; depth=96.39; mut=-	Y; depth=238.8; mut=-	Y; depth=3167.36; mut=R90K	Y; depth=49.31; mut=G595_V596insG; S589G	Y; depth=94.3; mut=N87I	Y; depth=1240.89; mut=-
CKIN62	Neg	N	5942.78	Y; depth=1991.2; mut=-	Y; depth=62.92; mut=-	Y; depth=40.78; mut=-	Y; depth=2739.9; mut=-	N; depth=0; mut=-	Y; depth=54.76; mut=-	Y; depth=1053.22; mut=-
CKIN63	Neg	N	24763.45	Y; depth=19370.95; mut=-	Y; depth=136.65; mut=-	Y; depth=74.4; mut=-	Y; depth=3501.09; mut=-	N; depth=1.9; mut=-	Y; depth=36.63; mut=N87T	Y; depth=1643.72; mut=-

CKIN64	Neg	N	5400.76	Y; depth=1428.09; mut=-	Y; depth=104.95; mut=-	Y; depth=128.04; mut=-	Y; depth=2383.09; mut=-	N; depth=0.79; mut=-	Y; depth=40.41; mut=-	Y; depth=1316.18; mut=-
CKIN67	Neg	N	2125.84	Y; depth=1061.34; mut=insG927GC	Y; depth=26.88; mut=-	Y; depth=50.19; mut=-	Y; depth=987.43; mut=-	N; depth=1; mut=-	N; depth=0; mut=-	N; depth=1; mut=-
CKIN7	Neg	Y	1655.64	Y; depth=736.05; mut=-	Y; depth=58.56; mut=A2143G	Y; depth=218.77; mut=-	Y; depth=103.3; mut=R90K	Y; depth=244.68; mut=S589G	Y; depth=182.53; mut=-	Y; depth=111.75; mut=-
KIN1	Neg	Y	2148.54	Y; depth=917.96; mut=-	Y; depth=54.57; mut=A2143G	Y; depth=194.05; mut=-	Y; depth=262.12; mut=R90K	Y; depth=257.19; mut=S589G	Y; depth=259.23; mut=-	Y; depth=203.42; mut=-
KIN2	Neg	Y	1836.18	Y; depth=797.09; mut=-	Y; depth=40.36; mut=A2143G	Y; depth=182.16; mut=-	Y; depth=180.74; mut=R90K	Y; depth=212; mut=S589G	Y; depth=201.2; mut=-	Y; depth=222.63; mut=-

Notes: Loci with sequencing depth ≤2 reads were considered absent. 'Global targeted-loci read depth' is used as a proxy for relative *H. pylori* DNA signal and does not establish bacterial viability or absolute abundance. Gene cells summarize detection (Y/N), depth, and AMR-associated mutation(s) where observed.

Supplementary Table S4. Per-sample FASTQ file sequencing yield and *H. pylori* mapping statistics from two combined sWGA sequencing runs

**Sample**	**Run1 raw reads**	**Run2 raw reads**	**raw bp Run1**	**Raw bp Run2**	**Total reads Run1&2**	**total_bp Run1&2**	**Hp mapped reads**	**Hp mapped bp**
CKIN7	29059	4488	124156257	27810224	33547	151966481	366	876676
CKIN16	14330	2306	44988019	9542232	16636	54530251	326	779664
CKIN20	8510	1599	25263258	5691602	10109	30954860	293	721797
CKIN18	34011	4679	138537710	29199778	38690	167737488	317	846476
CKIN21	70703	8168	278338599	58076702	78871	336415301	376	1078925
CKIN23	49429	8093	294926932	73682132	57522	368609064	240	657032
CKIN13	39390	5123	146473220	31661771	44513	178134991	316	811633
CKIN19	138293	19105	603339267	137300648	157398	740639915	440	1290410
KIN1	215798	18713	660085823	123399985	234511	783485808	410	1123261
CKIN11	38066	5028	174583020	38585960	43094	213168980	200	611901
KIN2	79649	11025	396703269	91801455	90674	488504724	335	1037364

Only a small proportion of reads mapped to *H. pylori*, consistent with low biomass. Variations in mapped reads reflect differences in recovered sequencing signals and, due to sWGA biases (uneven amplification and preferential enrichment), suggest partial and inconsistent genome recovery rather than the true absence of bacterial DNA.

Supplementary Table S5. Recovery metrics of *H. pylori* fragment sequences from *H. pylori* phenotypically negative gastric biopsies using the DFAST platform.

Sequences submitted to DFAST are mapped reads converted to FASTA format and do not represent assembled genomes.

Sample	GC content (%)	N50	Organism	ANI (%)	Completeness (%)	Contamination (%)
CKIN7	39.4	4,218	*H. pylori*	92.3	18.7	10.0
CKIN16	39.2	4,481	*H. pylori*	91.7	16.6	6.9
CKIN20	39.3	4,570	*H. pylori*	93.8	16.0	11.0
CKIN18	40.4	4,786	*H. pylori*	93.5	15.4	4.9
CKIN21	40.7	5,301	*H. pylori*	92.1	16.6	6.1
CKIN23	41.4	5,431	*H. pylori*	93.2	12.0	4.5
CKIN13	39.8	5,454	*H. pylori*	92.8	13.0	6.7
CKIN19	42.0	5,560	*H. pylori*	93.1	24.0	10.7
KIN1	43.0	5,638	*H. pylori*	92.2	21.0	9.7
CKIN11	41.0	6,416	*H. pylori*	92.1	11.0	4.6
KIN2	44.4	8,383	*H. pylori*	92.1	14.6	9.6

Metrics from sWGA-derived, read-enriched *H. pylori* sequence fragments showed lower completeness and higher fragmentation across samples, which were expected due to selective amplification and partial recovery from low-biomass biopsies. Therefore, the non-recovery of specific loci was not considered true genomic absence. Despite variability in fragment yield and completeness estimates, ANI values consistently confirmed *H. pylori* assignment, indicating the presence of recoverable *H. pylori* DNA fragments even in low-abundance samples.

Supplementary Table S6. Primers used for PCR amplification of antimicrobial resistance genes in *H. pylori*

	Primer Name	Primer type	Sequence (5' - 3')	Amplicon length (bp)	Gene length (bp)
1	RdxA.F	Forward	GACAATTACTGAACGAGCGCCATTCTTG	~550	~633
2	RdxA.R	Reverse	GCCACCCTCTTACCCAAAGCG		
3	FrxA.F	Forward	GCTTTACAGCACCAACGATTCGCTAC	~600	~654
4	FrxA.R	Reverse	CCAGCGGGTTTTAGGGGTGATTTC		
5	16S-rRNA.F	Forward	GTTTGATCCTGGCTCAGAGTGAACGC	~1494	~1509
6	16S-rRNA.R	Reverse	GAAAGGAGGTGATCCAACCGCAGG		
7	23S-rRNA.F	Forward	GAGAGGCGATGAAGGACGTACTAGACTG	~2731	~2967
8	23S-rRNA.R	Reverse	CTTGGAGTTGGCTTCCTGCTTAGATGC		
9	gyrA.F	Forward	GGCTTATTCCATGAGCGTGATCATAGGG	~1663	~2487
10	gyrA.R	Reverse	CCGGCTCGCTTCTGGGATTTTATAGAC		
11	gyrB.F	Forward	CACCACATGGTGTATGAAGTCGTGGAT	~2171	~2322
12	gyrB.R	Reverse	CTTCTAGGCTCTACTTCATCGCCCATG		
13	pbp1A.F	Forward	GCCATTCTTATCGCTCAAGTTTGGGTAAC	~1918	~1980
14	pbp1A.R	Reverse	AGAACAACAAGCGTTCTTCGCTATCGTCTG		

Primers were designed from full-length consensus sequences obtained by aligning antimicrobial resistance-associated genes from African *H. pylori* genomes ARGs using SnapGene v7.2.1 (Dotmatics, Boston, MA, USA). Targeted loci included gyrA and gyrB (fluoroquinolone resistance), 16S rRNA (tetracycline resistance), 23S rRNA (macrolide resistance), frxA and rdxA (metronidazole resistance), and pbp1A (amoxicillin resistance). Amplicon sizes correspond to expected PCR products based on the reference genome *H. pylori* strain 26695.

Supplementary Table S7. In silico evaluation of primer binding and predicted amplification efficiency for *H. pylori* antimicrobial resistance genes

Primer	Length (bp)	85% of length	Minimum alignment threshold	Gene	genomes analyzed	Perfect paired matches	predicted amplification
RdxA.F	28	23.8	24	*rdxA*	197	53 (26.9%)	
RdxA.R	21	17.85	18				196 (99.5%)
FrxA.F	26	22.1	23	*frxA*	218	84 (38.5%)	118 (54.1%)
FrxA.R	24	20.4	21				
16S-rRNA.F	26	22.1	23	*16S-rRNA.F*	287	287 (100%)	287 (100%)
16S-rRNA.R	24	20.4	21				
23S-rRNA.F	28	23.8	24	*23S-rRNA.F*	279	279 (100%)	279 (100%)
23S-rRNA.R	27	22.95	23				
gyrA.F	28	23.8	24	*gyrA*	271	147 (54.2%)	247 (91.1%)
gyrA.R	27	22.95	23				
gyrB.F	27	22.95	23	*gyrB*	292	251 (86%)	292 (100%)
gyrB.R	27	22.95	23				
pbp1A.F	29	24.65	25	*pbp1A*	316	84 (26.6%)	242 (76.6%)
pbp1A.R	30	25.5	26				

An in silico primer coverage analysis was conducted on African *H. pylori* genomes used for primer design. Perfect paired primer matches ranged from 26.6% to 100% depending on the locus, reflecting sequence variation within primer-binding regions. However, allowing up to 3 mismatches and alignment at least 85% of the primer length, predicted amplification rates ranged from 54.1% to 100%, indicating that most genomes are likely to remain amplifiable despite primer mismatches.

<u>Bioinformatics script commands for paired matches and predicted amplification counts.</u>

<u>Perfect paired matches count</u>

For instance, for gyrB

```
awk 'BEGIN{FS=OFS="\t"}
$1=="gyrB_F" && $3==100 && $4==27 && $5==0 {F[$2]=1}
$1=="gyrB_R" && $3==100 && $4==27 && $5==0 {R[$2]=1}
END{
  c=0
  for (g in F) if (g in R) c++
  print c
}' gyrB_primer_matches.txt
```

<u>Count predicted amplification</u>

The definition of predicted amplification is as:

forward and reverse both present
alignment length at least 85% of primer length,
mismatches ≤ 3

For instance, for gyrA:

forward threshold: >= 24 for a 28 bp primer
reverse threshold: >= 23 for a 27 bp primer

We have used:

```
awk 'BEGIN{FS=OFS="\t"}
$1=="gyrA_F" && $4>=24 && $5<=3 {F[$2]=1}
$1=="gyrA_R" && $4>=23 && $5<=3 {R[$2]=1}
END{
  c=0
  for (g in F) if (g in R) c++
  print c
}' gyrA_primer_matches.txt
```

Supplementary Figure S1. Global ARGs sequence depth in gastric biopsies from phenotypically negative and positive patients.

Values indicate locus-specific read depth measured under consistent amplification and sequencing conditions. Variations show differences in recovered sequencing signal and should not be viewed as exact gene abundance.

Supplementary Figure S2. Growth dynamics of *H. pylori* during natural transformation and clarithromycin selection (D1-D3).

H. pylori growth was scored daily from Day 1 (D1) to Day 3 (D3) based on visual inspection of colony outgrowth. Plates were inoculated in duplicate for each condition.
(A) Clarithromycin selection. No colonies grew in negative controls (recipient strain 26695 without donor DNA and transformation with the wild-type *23S rRNA* amplicon from *H. pylori* 26695), regardless of clarithromycin concentration. Transformation with the A2143G *23S rRNA* amplicon from the CKIN12_pos biopsy resulted in earlier and higher colony growth than transformation with the A2143G *23S rRNA* amplicon from CKIN7_neg. For CKIN12_pos, mean colony counts showed early outgrowth by D2 at 0.25 and 0.5 µg/mL (means approximately 4.25 and 0.5, respectively) and strong growth by D3 across 0.25–4 µg/mL (means approximately 314.25, 273.5, 212.75, 195.25, and 159.5 at 0.25, 0.5, 1, 2, and 4 µg/mL, respectively). For CKIN7_neg, colony outgrowth was more moderate on D3 (means approximately 135.5, 54, 49.5, 29.5, and 5 at 0.25, 0.5, 1, 2, and 4 µg/mL, respectively).

(B) Antibiotic-free conditions. Growth on clarithromycin-free plates was scored as follows: *no growth* (no detectable colonies), *merging colonies* (patchy colony expansion), and *thick mat* (dense confluent biomass). Under antibiotic-free conditions, cultures exposed to donor DNA (CKIN12_pos and CKIN7_neg) and the wild-type *H. pylori* 26695 control showed rapid expansion from D2 onward, while the medium-only control (Brucella broth supplemented with 10% fetal bovine serum) remained negative throughout D1-D3. These observations suggest that transformant-associated cultures did not exhibit an **obvious growth defect** under antibiotic-free conditions; however, formal fitness effects were not quantified in this study.

Supplementary Figure S3. Mapping of *23S rRNA* SNPs in *H. pylori* recipient, donor (CKIN12), and transformant strains.

This figure summarizes single-nucleotide polymorphisms (SNPs) across 23S rRNA gene sequences from the *H. pylori* recipient strain 26695, the donor sample CKIN12 (phenotypically positive for *H. pylori*), and 11 independent transformants. SNPs were visualized using Snipit from multiple sequence alignments. SNP coordinates are shifted by +5 bp relative to conventional *23S rRNA* annotation due to alignment offsets caused by insertions/deletions (indels) at positions 241, 764, and 1943 in the recipient sequence. Accordingly, the canonical clarithromycin-resistance marker A2143G is displayed here as A2148G.

The dataset includes both copies of the *23S rRNA* gene from the recipient strain 26695 obtained through whole-genome sequencing (WGS), a consensus *23S rRNA* sequence from donor CKIN12 generated by targeted PCR sequencing, and both *23S rRNA* gene copies from 26695 transformants obtained through WGS after selection on clarithromycin (4 µg/mL). Strain

nomenclature follows: [Background strain]-[target gene][mutation]-[donor sample]-[selection condition]-[Clone#]. Notably, natural transformation–mediated homologous recombination introduced the A2143G mutation (displayed here as A2148G) into both *23S rRNA* gene copies in all transformants except 07t, where it was detected in only one copy. Additional *23S rRNA* SNPs were observed in some transformants; none correspond to previously known clarithromycin-resistance markers.
