# Supplementary figures and images for: Low-Abundance and Fragmentary Helicobacter pylori DNA Detected in Phenotypically Negative Gastric Biopsies Using Targeted Sequencing
Source: Biomolecules. 2026 May 22;16(6):765. doi: 10.3390/biom16060765 (PMC13296529; doi:10.3390/biom16060765)

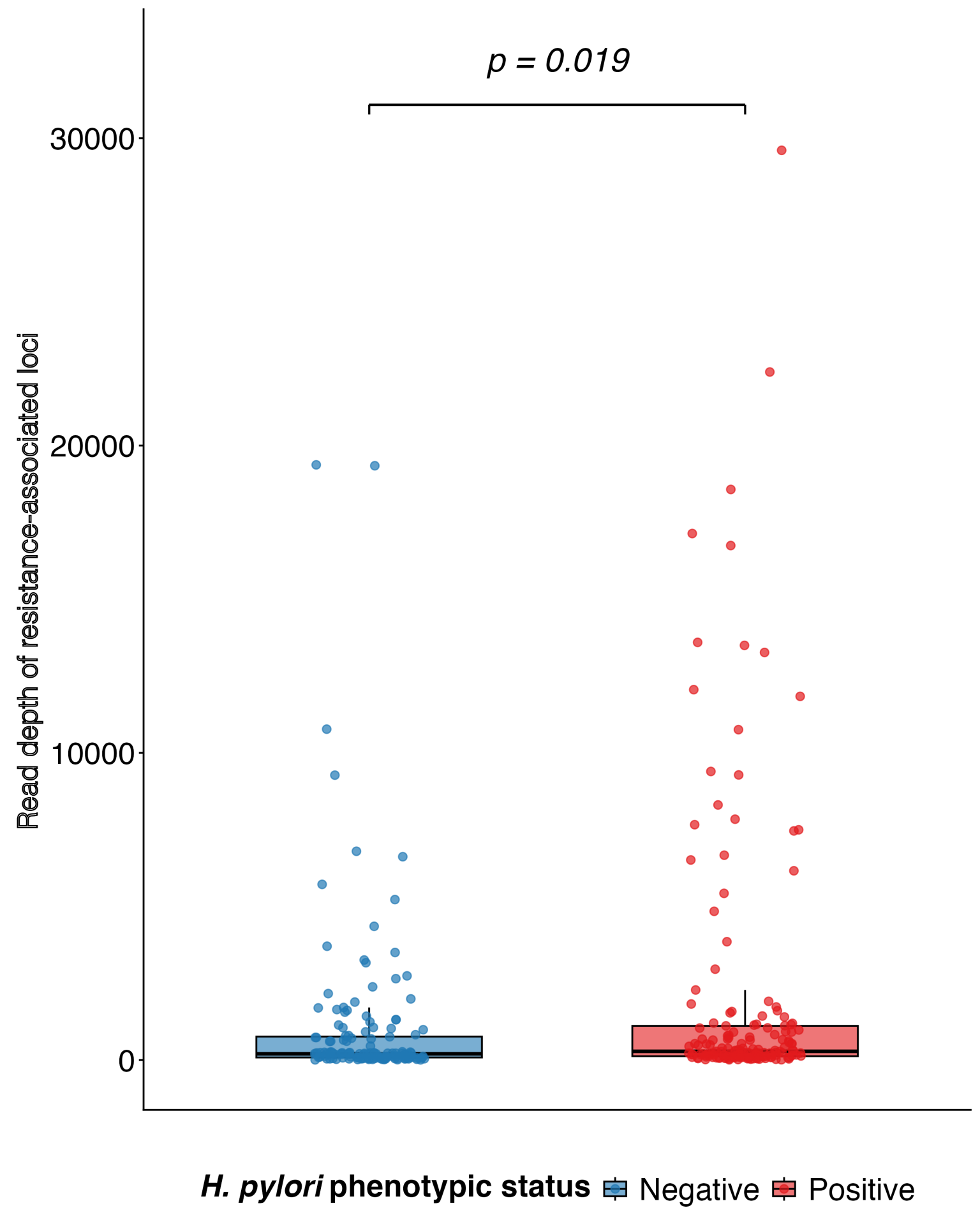

Supplement: Supplementary file 1 [file biomolecules-16-00765-s001.zip › Supplementary Figure S1.pdf]

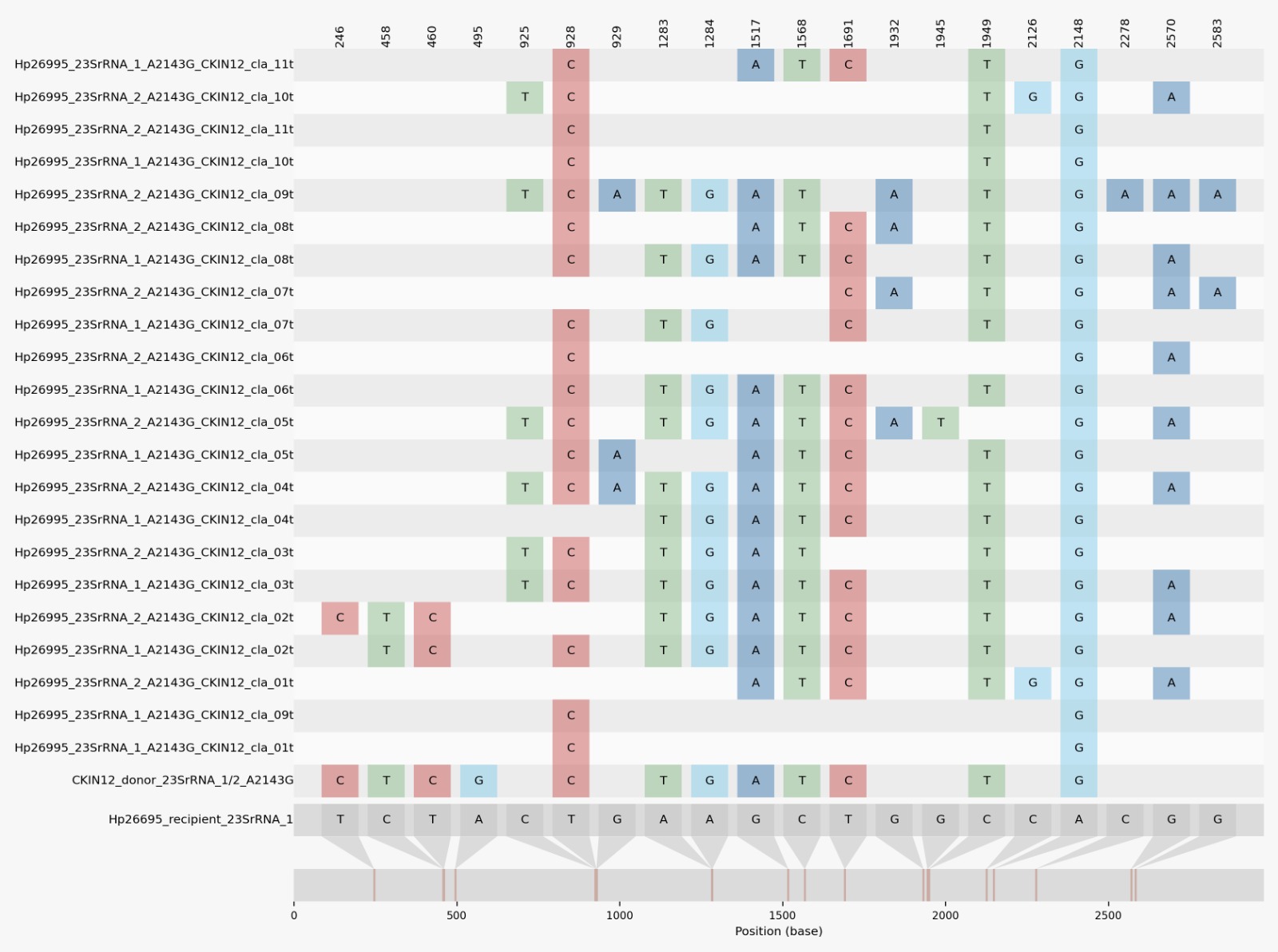

Supplement: Supplementary file 1 [file biomolecules-16-00765-s001.zip › Supplementary Figure S3.jpg]

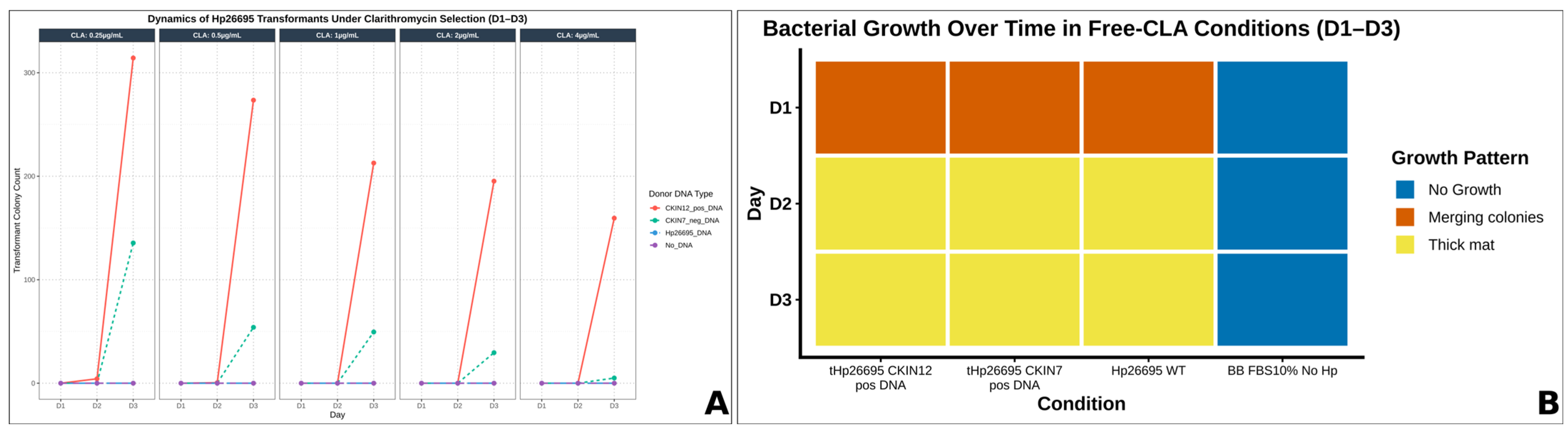

Supplement: Supplementary file 1 [file biomolecules-16-00765-s001.zip › Supplementary_Figure_S2.tiff]
